# Sticky Architecture: Encoding Pressure Sensitive Adhesion in Polymer Networks

**DOI:** 10.1021/acscentsci.2c01407

**Published:** 2023-02-01

**Authors:** Mitchell Maw, Erfan Dashtimoghadam, Andrew N. Keith, Benjamin J. Morgan, Alexander K. Tanas, Evgeniia Nikitina, Dimitri A. Ivanov, Mohammad Vatankhah-Varnosfaderani, Andrey V. Dobrynin, Sergei S. Sheiko

**Affiliations:** † Department of Chemistry, University of North Carolina at Chapel Hill, Chapel Hill, North Carolina 27599-3290, United States; ‡ Lomonosov Moscow State University, Leninskie Gory 1, 119991, Moscow, Russian Federation; § Institut de Sciences des Matériaux de Mulhouse-IS2M, CNRS UMR 7361, 15, rue Jean Starcky, F-68057 Mulhouse, France

## Abstract

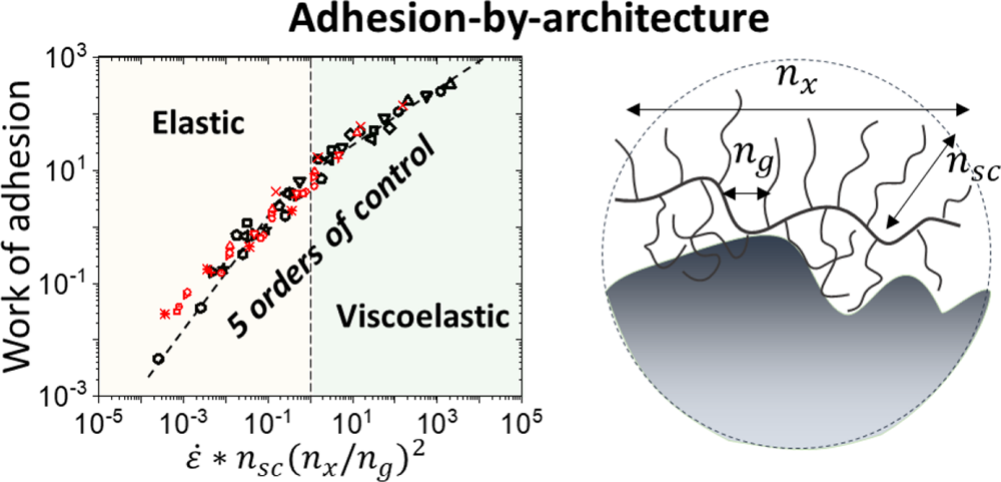

Pressure sensitive adhesives (PSAs) are ubiquitous materials within a spectrum that span from office supplies to biomedical devices. Currently, the ability of PSAs to meet the needs of these diverse applications relies on trial-and-error mixing of assorted chemicals and polymers, which inherently entails property imprecision and variance over time due to component migration and leaching. Herein, we develop a precise additive-free PSA design platform that predictably leverages polymer network architecture to empower comprehensive control over adhesive performance. Utilizing the chemical universality of brush-like elastomers, we encode work of adhesion ranging 5 orders of magnitude with a single polymer chemistry by coordinating brush architectural parameters–side chain length and grafting density. Lessons from this design-by-architecture approach are essential for future implementation of AI machinery in molecular engineering of both cured and thermoplastic PSAs incorporated into everyday use.

## Abstract 2

Brush architecture allows varying the work of adhesion within many orders of magnitude without using additives and altering chemical composition.

## Introduction

**Figure 1:**
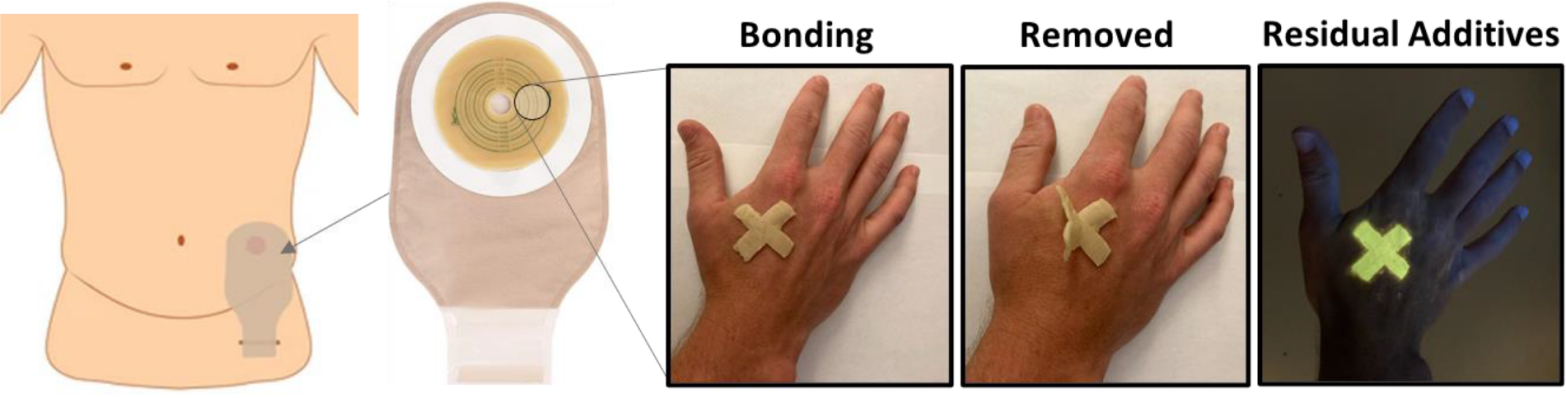
Composite PSA design entails hazardous leaching and property drift. A commercial ostomy adhesive was formed into an X shape and applied to skin. After 30 min, the adhesive was removed. Though invisible to the naked eye, residual additives were left on the skin at risk of penetrating an open wound. For clear observation of the migratory components, a fluorescent powder was spread over the skin surface where it adhered to the residue and was revealed by a black light.

Adhesives fall into two main categories: structural adhesives, such as glues, and pressure sensitive adhesives (PSAs), such as mounting tapes, both relying on large interfacial contact area to bond with a substrate.^1,2^ Structural adhesives achieve interfacial contact by administering fluid resins that readily wet surface pores and subsequently cure, permanently binding to the substrate.^3^ However, many adhesive applications require removability or merely prohibit the use of fluids. These issues are addressed by the employment of solid PSAs where the contact area increases over time through viscoelastic compliance under applied pressure.^4^ The range of viscoelastic behaviors for these materials encompasses a diverse class of adhesives, from easily removable Post-it notes to shear resistant ostomy bags.^5−9^ Current approaches to navigate through this wide property space rely on the exploratory mixing of polymer networks with additives, such as tackifiers and plasticizers, which entails property drift and surface contamination due to chemical migration (Figure 1).^1,10−14^ This presents a challenge to develop an alternative route to regulate material viscoelasticity without using additives and altering chemical composition.

**Figure 2:**
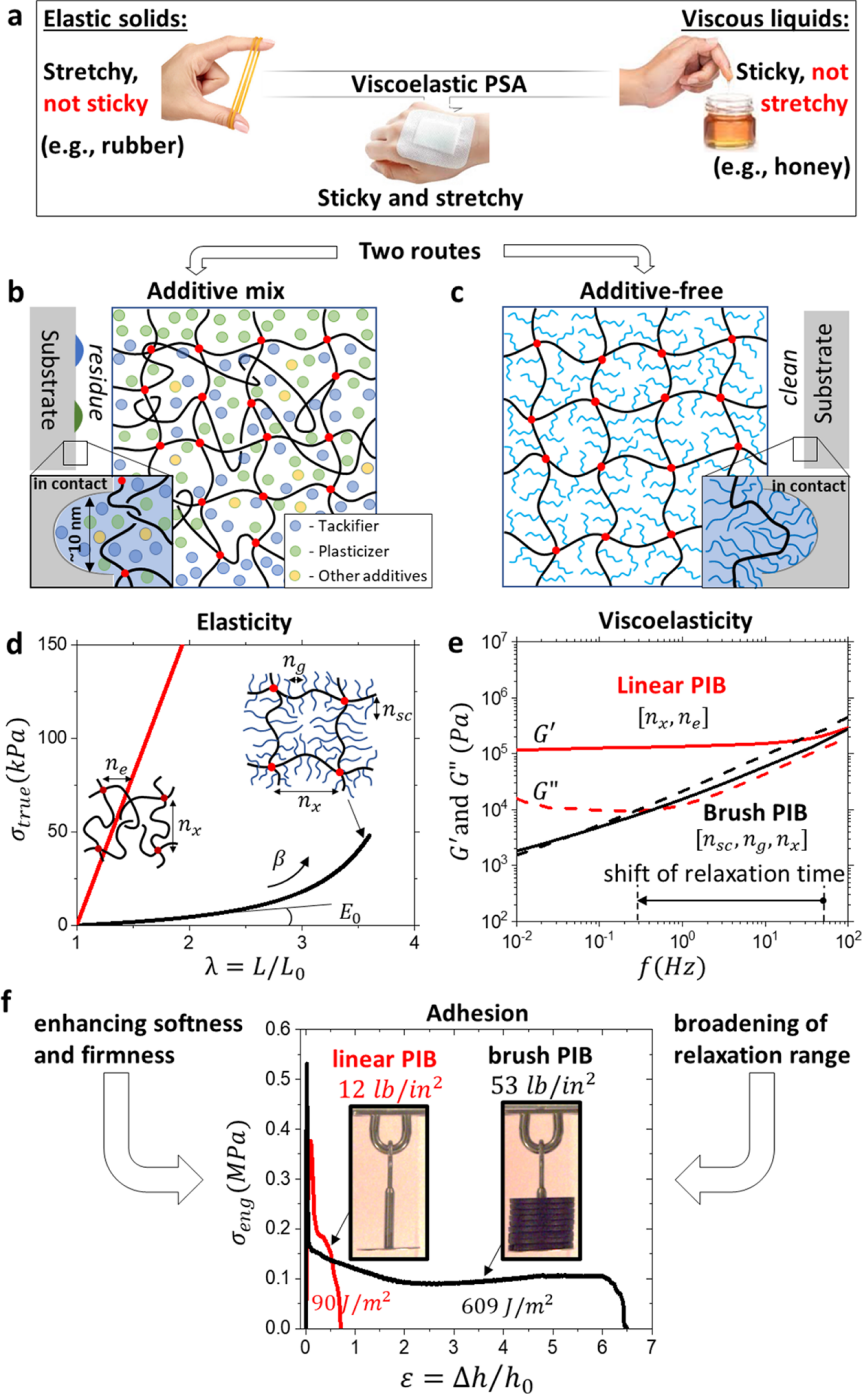
Additive-free control of adhesive performance. (a) Pressure sensitive adhesives integrate the elasticity of rubber and the tackiness of viscous liquids, which can be implemented in two orthogonal ways (b, c). (b) Linear polymer networks require large quantities (up to 50 wt %) of loose additives to dilute chain entanglements and facilitate substrate wetting, resulting in surface residues upon debonding. The high entropic penalty of dual-end constrained linear strands hinders their penetration into nanoscopic cavities forcing reliance on diluents to wet the surface without the ability to transfer stress (inset). (c) Grafted side chains act as nonleachable diluents of chain entanglements and concurrently promote nanoscale wetting with minimal entropy loss due to redistribution of the side chain ends (inset). (d) Stress–elongation curves (ε̇ = 0.005 s^–1^) of exemplary linear (red) and brush (black) polyisobutylene (PIB) elastomers. Even though both networks have similar degrees of polymerization (DP) between cross-links *n*_x_ ≅ 300, brush elastomers are considerably softer and exhibit much stronger strain-stiffening, characterized by parameter β = ⟨*R*_in_^2^⟩/*R*_max_^2^, ratio of the mean square distance of network strands to the square of their contour length.^29^ The modulus of linear networks is controlled by chain entanglements characterized by the entanglement DP *n*_e_ ≅ 150, whereas architecturally disentangled brush networks extend their softness through side chains of DP *n*_sc_ and grafting density *n*_g_^–1^. (e) Frequency sweeps of the storage (*G′*, solid lines) and loss (*G*″, dashed lines) moduli for the linear and brush PIB elastomers from (d). Linear chain networks demonstrate the elastic response defined by an entanglement plateau modulus of *G*_e_ ≈ 10^5^ Pa. In contrast, side chains in brush elastomers lower the modulus and extend the window of viscoelastic response toward the PSA frequency range. (f) Adhesive stress (*σ*_eng_) as a function of pull-off strain (ε) for the linear and brush PSAs from (d, e) measured by probe tack testing (ε̇ ≈ 1 s^–1^) and hanging loads (insets) at 20 °C where ε is defined by the ratio of pull-off distance Δ*h* to initial PSA thickness *h*_0_. Linear PIB witnesses minimal tack and work of adhesion (*W*_adh_), as indicated under the red curve, and is unable to uphold a load above 12 lb/in^2^. The ability of brush architecture to concurrently regulate elastic (softness and firmness) and viscoelastic (relaxation time) properties results in greater tack and *W*_adh_, as well as the ability to withstand a hanging load of 53 lb/in^2^ for sample [*n*_sc_ = 18, *n*_g_ = 1, *n*_*x*_ = 300]. The work of adhesion is determined as *W*_adh_ = *h*_0_ ∫_0_^*ε*_max_^*σ*_eng_(ε) d*ε*, where *ε*_max_ is the maximum strain before bond failure.

Viscoelasticity defines both bonding behavior under pressure and PSA deformation upon debonding accompanied by cavitation and fibrillation processes.^15−19^ The bonding-debonding dualism makes optimization of performance especially challenging, as it mandates a convolution of oxymoronic properties. PSAs should be sticky like viscous liquids, yet removable like elastic solids to leave a clean surface after debonding (Figure 2a). They should also be simultaneously soft and strong to facilitate substrate wetting upon bonding and withstand cohesive rupture upon debonding, respectively.^20^ Additionally, bonding and debonding typically occur at different time scales—slow bonding and much faster peel off, which imposes specific requirements for frequency dependence on the storage and loss moduli within the PSA-relevant frequency range of 0.01 to 100 Hz.^21^ Current PSA designs manage this interplay of conflicting demands through controlled mixing of polymers with specific types of low molecular weight additives. Polymer networks alone are too stiff due to chain entanglements (*G*_e_ ≈ 10^5^ Pa),^22^ so large quantities (up to 50 wt %) of tackifiers and plasticizers are required to dilute the network strands and satisfy the Dahlquist criterion (shear modulus *G* < 10^5^ Pa) for spontaneous wetting of the surface roughness (Figure 2b).^1,23^ However, additives are prone to migration and trigger considerable shifts in the modulus frequency spectrum, which may alter the debonding behavior or completely forfeit adhesion.^1,10^ Moreover, balancing the conflicting effects in multicomponent materials is an arduous feat with limited resources for property precision, predictability, and stability over time, which has inspired the search for additive-free alternatives.^24,25^

We have developed an alternative approach to PSA design by introducing brush architecture into network strands (Figure 2c). This empowers encoding of viscoelastic properties without changing the chemical composition or using additives. In particular, tethered side chains facilitate both bonding and debonding on different length and time scales. During bonding, side chains effectively play the role of diluents that disentangle brush backbones to decrease the Young’s modulus (*E*_0_) from 10^5^ to 10^2^ Pa and promote wetting of microscopic pores.^26,27^ In addition, the free-ended side chains experience less entropic penalty upon wetting of nanoscopic cavities than dual-end anchored linear strands further increasing the effective contact area (Figure 2b,c insets). In the case of debonding, steric repulsion between the densely grafted side chains enhances strain-stiffening (β = 0.01–0.2),^28,29^ which in turn promotes cohesive strength (Figure 2d). In parallel with the elastic response, brush architecture fundamentally changes the network viscoelastic profile by shifting both the characteristic relaxation times (e.g., Rouse time, *τ*_R_) and the entanglement plateau to ensure concurrent increase of the storage and loss moduli within the PSA frequency range (Figure 2e).^30,31^ By independently controlling elastic and viscoelastic properties (Figure 2f), we demonstrate the utility of brush elastomers for a range of applications from removable adhesives to high shear PSAs, which span 5 orders of magnitude of the overall work of adhesion (*W*_adh_) for chemically identical materials as discussed below. This approach enables (i) single component nonleaching materials, (ii) a wide property range for a given chemistry, (iii) precise property control, (iv) the ability to adjust elastic and viscoelastic properties independently, and (v) stability of the adhesive performance over time. This is achieved by directly linking *W*_adh_ and tack stress (*σ*_tack_) to macromolecular architecture through the relaxation dynamics of brush polymer networks. Furthermore, this strategy can be advanced to physically cross-linked networks, such as thermoplastic elastomers, for hot-melt PSA molding and 3D printing.

## Results and Discussion

To elucidate the effect of architecture on adhesion, we prepared a broad series of brush networks with systematically varied side chain degrees of polymerization (DP), *n*_sc_, and grafting densities, *n*_g_^–1^, (Table S1) spanning different brush conformation regimes from comb to bottlebrush. To demonstrate universality of the architectural control, we synthesized two series of chemically different elastomers with poly(*n*-butyl acrylate) (PBA) and polyisobutylene (PIB) side chains (Figures S1–S7, S11–S13). These brush networks are well-defined with uniform mesh distribution achieved by relatively slow polymerization^28,29^ with the network structure verified by small-angle X-ray scattering (SAXS), where the distance between brush backbones correlates with grafting density and length of the side chains (Figure S14, Table S2).

**Figure 3:**
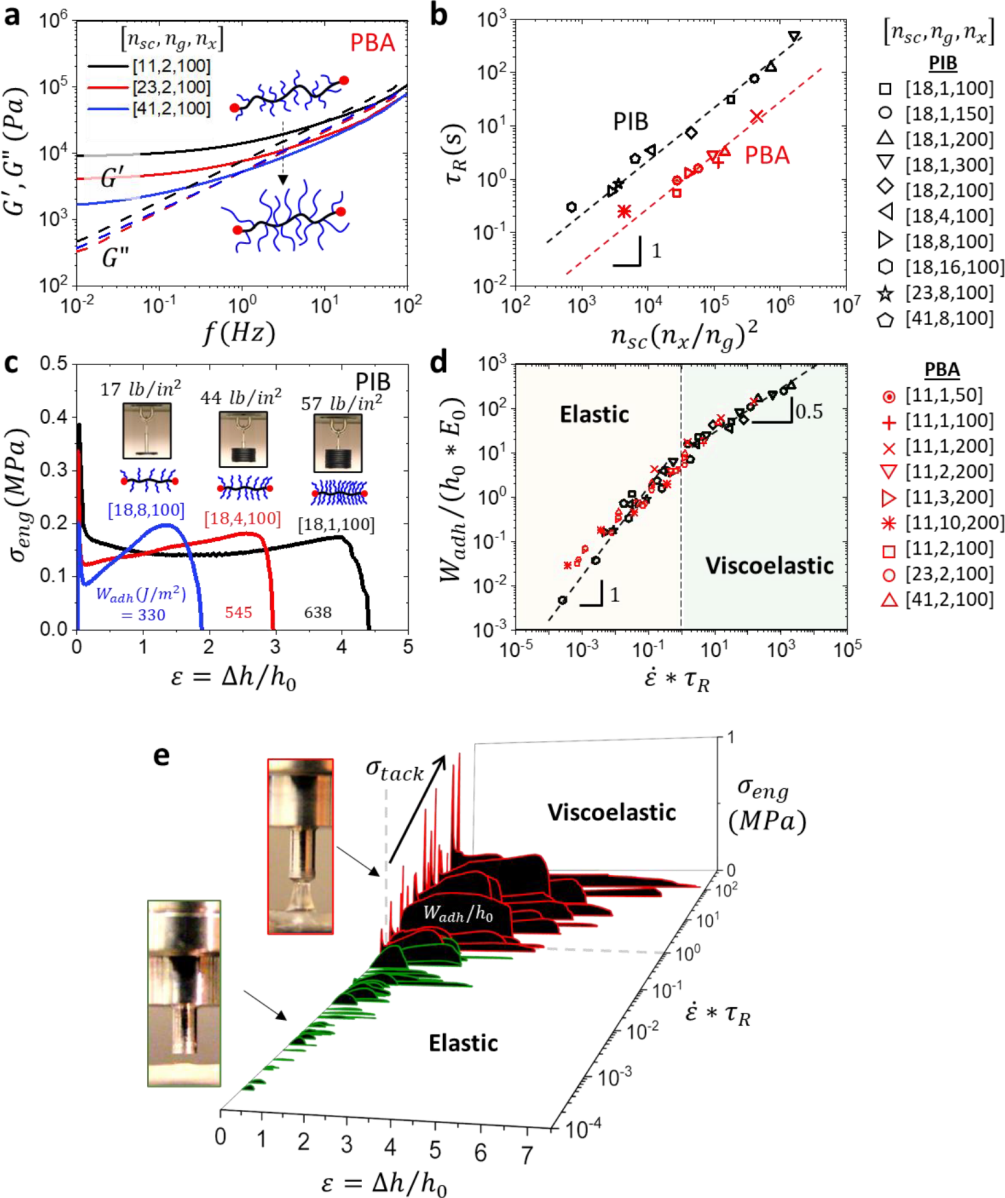
Mediating adhesion through architecturally controlled polymer relaxation. (a) Frequency sweeps of exemplary PBA brush elastomers display shifts in the viscoelastic spectrum by increasing the side chain length (∼*n*_sc_). The corresponding effects of *n*_g_ and *n*_x_ are shown in Figures S19–S24. The brush PSAs reach softness of *G′* ≈ 1 kPa, below the Dahlquist criterion, and simultaneously shift the onset of the elastic plateau to lower frequencies leading to variable stickiness (Movie S1). (b) Rouse times for PIB and PBA brush PSAs follow linear dependence on architecture according to eq 1 (Table S1, Figures S25–S29). The sample legend is displayed to the right of the plot for both (b) and (d). (c) Adhesive stress–strain curves for PIB brush elastomers with varying grafting density (∼*n*_g_^–1^) measured by probe tack testing (ε̇ ≈ 1 s^–1^, 20 °C). Increasing the grafting density (∼*n*_g_^–1^) leads to a concurrent increase in *σ*_tack_ and *W*_adh_. Insets: snapshots of hanging load tests just before debonding (Movie S2). (d) Normalized work of adhesion, *W*_adh_/(*h*_0_*E*_0_), as a function of the normalized strain rate, *ε̇τ*_R_, for PBA and PIB brush PSAs defines a strain rate-dependent shift from elastic to viscoelastic deformation. (e) An overlay plot of tack stress–strain curves at different strain rates from (d) displays the combined effect of the architecture (*τ*_R_) and strain rate (ε̇) on the deformation mechanism, *W*_adh_, and *σ*_tack_ of polymer networks. See Figure 2d for a brush network schematic including the corresponding architectural parameters. Brush PSAs enable the ability to scale debonding from elastic to viscoelastic mechanisms by changing architecture alone.

As noted above, viscoelastic properties of linear polymers are constrained by chain entanglements that set limits for both elastic modulus and the Rouse relaxation time of network strands as *G* > *G*_e_ and *τ*_R_ < τ_0_*n*_e_^2^, where *G*_e_ ≅ 0.1 MPa and *n*_e_ ≅ 100 are the entanglement modulus and DP of linear polymers, whereas τ_0_ is the characteristic relaxation time defined by monomer chemistry. We alleviate these restrictions by covalently attaching side chains to the strand backbone, which provides two vital benefits for PSA performance. First, side chains effectively dilute chain entanglements by increasing *n*_e_ ≈ 500–2000 (depending on the side chain length and grafting density), which in turn lowers the entanglement modulus to allow synthesis of ultrasoft networks (Figure 3a and Figures S15–S17).^26,27^ Second, the side chain parameters (*n*_sc_ and *n*_g_) provide additional degrees of freedom in controlling the relaxation times as
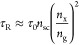
1(eq S14), allowing significant broadening of the Rouse regime with *τ*_R_ varying over 4 orders of magnitude (Table S1, Figures S19–S24). To quantify *τ*_R_ as an onset of Rouse relaxation, we conducted tensile tests in a broad range of strain rates (ε̇ = 10^–4^ - 10^1^ s^–1^) for PIB and PBA brush elastomers with systematically varied *n*_sc_ = 11–41, *n*_g_ = 1–16, and *n*_x_ = 50–300 (Figures S25–S29).^32^ For both brush chemistries, all architectures collapse on a respective single line according to eq 1 (Figure 3b), where the horizontal shift between the PIB and PBA lines is due to the change in polymer specific time scale τ_0_. In particular, τ_0_ is determined by the monomer projection length *l*, excluded volume *v*, Kuhn length *b*, and characteristic time scale of the segmental rearrangement controlling the monomeric friction coefficient ζ_0_ (see theoretical analysis in Supporting Information). The concomitant architectural regulation of both *G*_e_ and *τ*_R_ enables a two-decade increase in both storage (*G′*) and loss (*G*″) modulus within the PSA viscoelastic range meeting the requirements for softness, strength, and energy dissipation.

The effect of brush architecture on the adhesive performance is demonstrated by probe tack testing of the PIB and PBA elastomers at different debonding rates (Figures S30–S36).^19^ For example, decreasing grafting density at constant *n*_sc_ = 18 and *n*_x_ = 100 leads to a 2-fold increase in *W*_adh__,_*σ*_tack_, and *ε*_max_ (Figure 3c), which is consistent with the corresponding decrease of *G* and *τ*_R_ (Figure 3a,b, Figure S20). For a full range of the studied brush architectures and debonding rates, the architecture-controlled Rouse time allows for *W*_adh_ variation within 5 orders of magnitude (Figure 3d, Figure S35 and Tables S4–S6). It is important to emphasize that such large variations are achieved by tuning the architecture alone without altering chemical composition or using additives. Furthermore, all *W*_adh_ data points measured for the two chemically distinct brush PSA series at different strain rates (0.001–1 s^–1^) fall on a single line, which corroborates the universal nature of the adhesion-by-architecture approach. There is also an apparent switch from elastic to viscoelastic debonding mechanisms observed with increasing strain rate and identified by the slope change at ε̇ ≅ τ_R_^–1^. Below the Rouse rate (ε̇ < τ_R_^–1^), a given brush PSA debonds elastically through crack propagation along the surface where *W*_adh_/*E*_0_ ≈ *ε̇τ*_R_.^17,33^ At higher rates (ε̇ > τ_R_^–1^), debonding occurs in the viscoelastic regime through cavitation and fibrillation,^19,34^ where normalized work of adhesion scales as *W*_adh_/*E*_0_ ≈ (*ε̇τ*_R_)^1/2^. The ability to traverse from elastic to viscoelastic debonding through changes in a strand architecture is further corroborated by the emergence of the tack peak (Figure 3e), which corresponds to the onset of PSA yielding. The peak vanishes at ε̇ < τ_R_^–1^, where polymer chains have enough time to adjust to macroscopic deformation and maintain uniform stress distribution.

**Figure 4:**
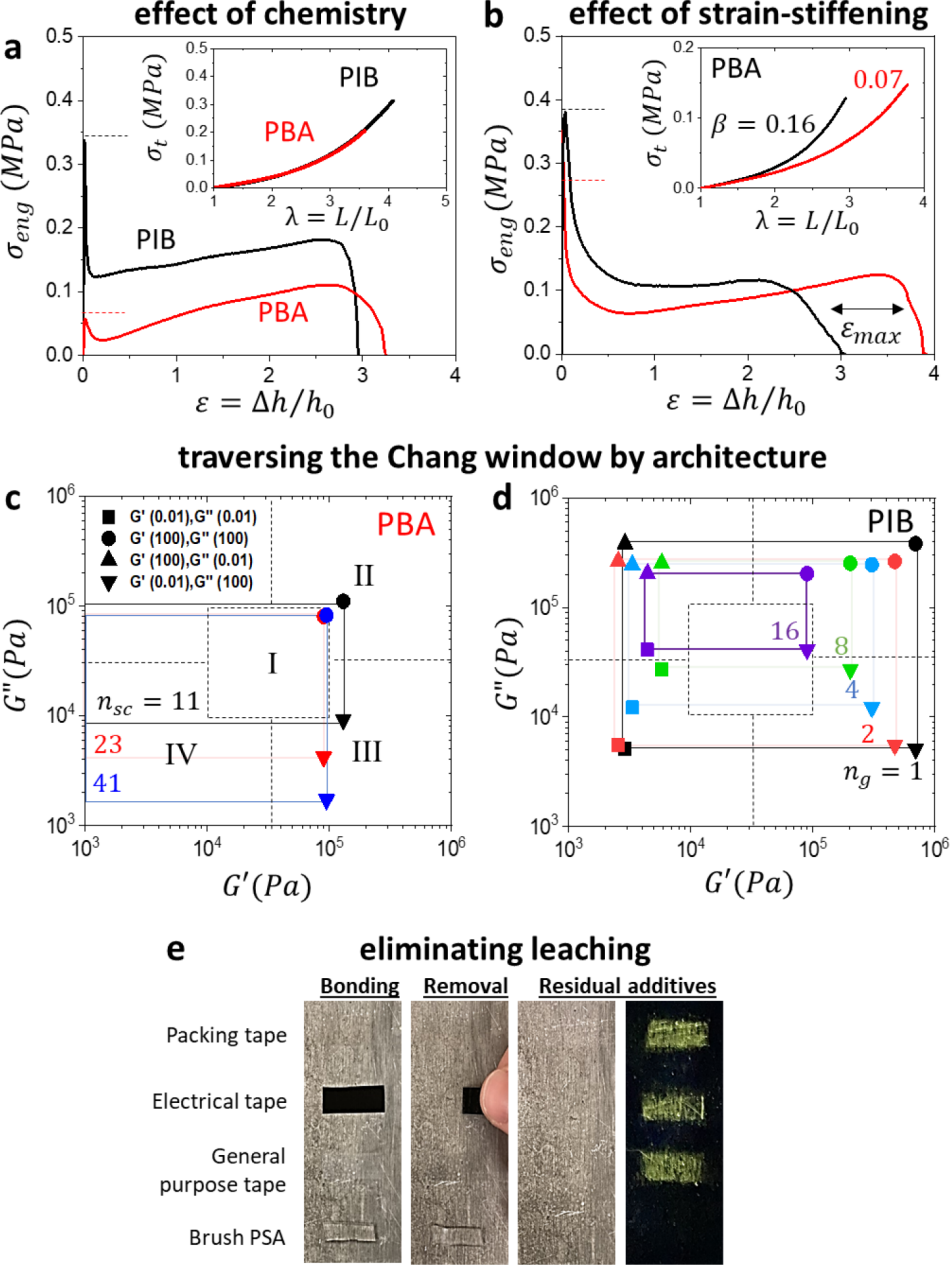
Versatility of brush PSAs in addressing the needs of specific applications. (a) Adhesive and tensile (inset) stress-deformation curves of brush elastomers with different chemistries. The PBA brush elastomer (red) with [*n*_sc_ = 11, *n*_g_ = 3, *n*_x_ = 200] and PIB brush sample (black) with [*n*_sc_ = 18, *n*_g_ = 4, *n*_x_ = 100] produce identical softness and strain-stiffening (*E*_0_ = 30 kPa, β = 0.08) but show different adhesion profiles. The PIB sample displays almost double the *W*_adh_ and a much larger tack peak (dashed lines) than the PBA sample (Movie S4) in agreement with the corresponding difference in their viscoelastic responses (Figure 3b, Figures S20 and S23). (b) Two samples of the same chemistry (PBA) yet different architectures, [*n*_sc_ = 11, *n*_g_ = 1, *n*_x_ = 100] (black) and [*n*_sc_ = 11, *n*_g_ = 2, *n*_x_ = 200] (red), reveal the effect of strain-stiffening on adhesion. The sample with larger β at the same *E*_0_ ≅ 20 kPa (inset) exhibits a decrease in maximum pull-off strain, *ε*_max_, while maintaining nearly the same *W*_adh_ ≅ 400 J*/*m^2^ (Movie S5). (c, d) Frequency sweeps of *G′* and *G′′* reveal the ability of brush elastomers to traverse the Chang window as exemplified by the variation in (c) side chain length (*n*_sc_ = 11–41) in PBA elastomers and (d) grafting density (*n*_g_ = 1–16) in PIB elastomers. The corners of the Chang window correspond to *G′* and *G′′* measured at 0.01 and 100 Hz (inset in panel c), where the highlighted rectangles correspond to I) classical adhesives like tapes, II) high shear resistant adhesives like mounting tapes, III) high peel resistant applications like labels, and IV) removable adhesives like protective films.^1^ (e) Acrylic and rubber-based commercial adhesives tapes leave residue on a substrate over time (18 h, 60 °C) revealed by fluorescent powder after tape removal (see Figure 1 for details). Brush PSAs contain no leachable additives, resulting in a clean surface after removal.

The wide-ranging control of material viscoelasticity at a given chemical composition without using additives empowers many benefits to adjust PSA performance for specific applications. For example, brush architecture permits control over the bulk deformation and adhesive response independently of one another. In Figure 4a, two brush PSAs of different chemistries (PIB and PBA) are architecturally programmed for almost identical nonlinear elastic response (*E*_0_ = 30 kPa and β ≈ 0.08) yet demonstrate considerably different *W*_adh_ values due to their distinct viscoelastic behaviors (Figures S20 and S23, Movie S3). Antithetically, the brush architecture of PIB and PBA samples can be adjusted to nearly identical adhesion with different elastic mechanical properties (Figure S37, Movie S4). The chemistry-independent control over the PSA performance is essential for applications that require a specific chemistry with desired thermomechanical stability, solvent resistance, or biocompatibility.

For a fixed chemistry, brush PSAs with the same softness for optimal bonding yet different strain-stiffening behaviors were prepared to control their debonding processes (Figure 4b, Movie S5). The firmer sample, β = 0.16, displays a larger *σ*_tack_, but lower *ε*_max_, resulting in nearly identical *W*_adh_ ≅ 400 J/m^2^. The intense strain-stiffening of bottlebrush PSAs prevents cohesive fracture upon debonding. Further tuning of performance by architecture is demonstrated by the ability to traverse the so-called Chang window, which acts as a map to identify specific PSA application areas (Figure 4c,d).^1,21^ And yet, all the studied additive-free brush PSAs do not leave residue over time or temperature variation upon removal, which contrasts with the behavior of commercial PSAs (Figure 4e).

**Figure 5:**
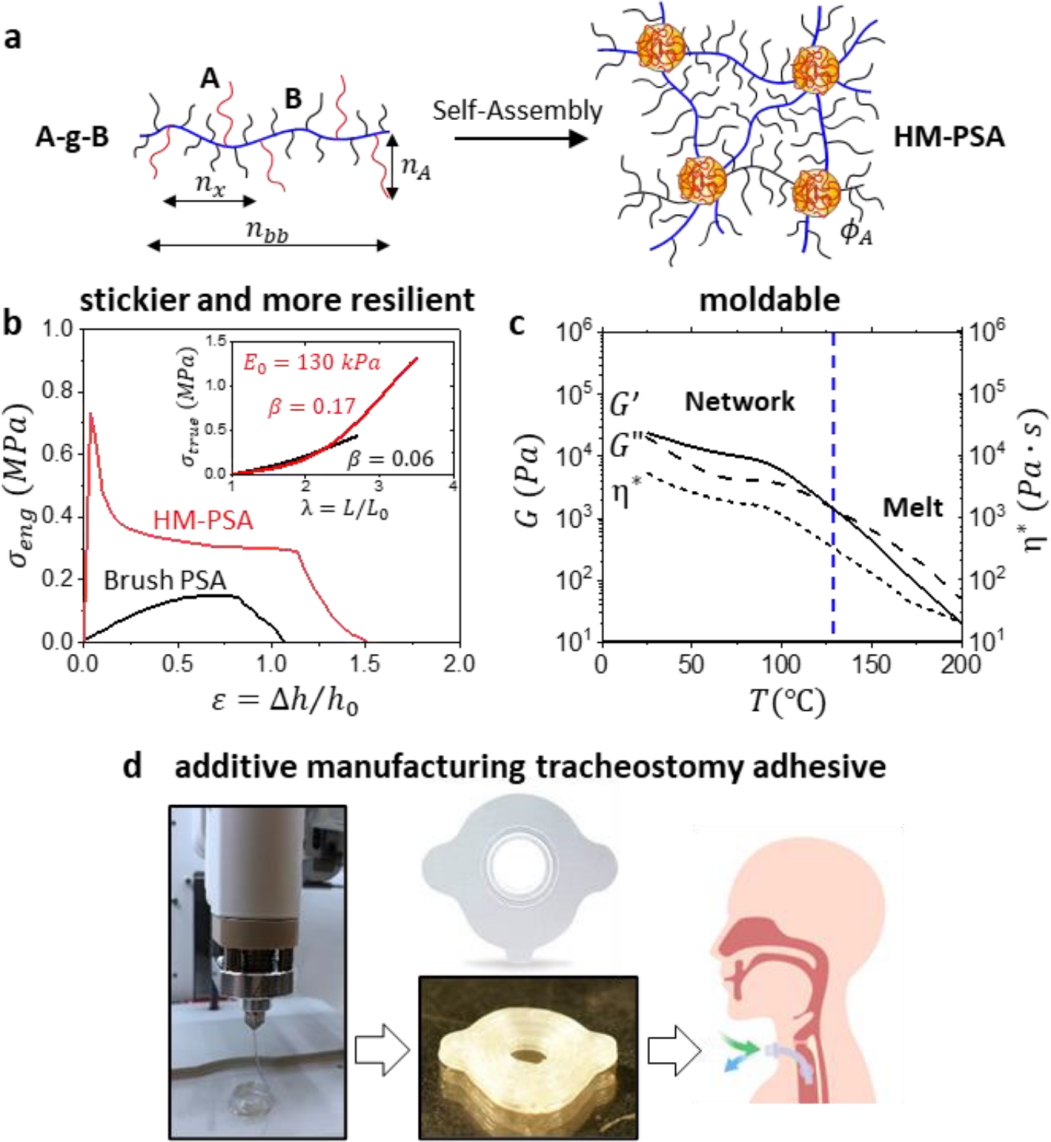
Expanding adhesion-by-architecture to hot-melt PSAs. (a) Brush-like graft block copolymers self-assemble into soft, firm, and strong physical networks. The A-*g*-B architecture enhances structural control of bottlebrush viscoelasticity by adding three parameters: number of A blocks per brush macromolecule *z* ≅ *n*_bb_/*n*_x_, DP *n*_A_ and volume fraction *ϕ*_A_ of A-blocks. (b) Adhesive stress–strain curves of an exemplary A-*g*-B copolymer (PS-*g*-PIB, *n*_sc_ = 18, *n*_g_ = 8, *n*_x_ = 332, *n*_A_ = 60, *ϕ*_A_ = 0.07) compared to a PIB brush elastomer (*n*_sc_ = 18, *n*_g_ = 16, *n*_x_ = 100) reveal a considerable difference between the self-assembled and covalent brush PSAs. The PS-*g*-PIB physical network with a similar Young’s modulus of *E*_0_ ≅ 130 kPa but greater firmness of β = 0.17 (inset) exhibits viscoelastic debonding with the emergence of a tack peak, while the brush elastomer undergoes elastic debonding with no tack and much lower *W*_adh_. (c) Temperature variation of the storage (*G′*) and loss (*G″*) moduli as well as complex viscosity (η*) of a PS-*g*-PIB sample (*n*_sc_ = 18, *n*_g_ = 8, *n*_x_ = 216, *n*_A_ = 60, *ϕ*_A_ = 0.1) displays network disassembly at 126 °C denoted as the temperature where *G″* surpasses *G′* (*f* ≈ 1 Hz, ε = 0.05). (d) Fused filament fabrication of the brush HM-PSA from (c) is used for 3D printing of a reduced scale tracheostomy adhesive (150 °C, see Supporting Information for printing parameters).

To demonstrate ubiquity in other systems, the brush platform was extended to the design of moldable PSAs, so-called hot-melt pressure sensitive adhesives (HM-PSAs). The moldability was introduced by replacing covalent cross-links with microphase separation in brush-like graft block copolymers denoted as A-*g*-B, where a controlled fraction of long A blocks were grafted along a bottlebrush B block of a different chemical composition (Figure 5a, Figures S8–S10).^35^ Specifically, A-*g*-B’s with polystyrene (PS) grafts (A blocks) of DP *n*_A_ and bottlebrush blocks with PIB side chains of DP *n*_sc_ undergo microphase separation to produce a physical network linked by A-block domains. Phase separation in block copolymers is dependent on dimensions of the constituting blocks, creating a narrow window for the desired viscoelastic response of PSAs. For example, above *ϕ*_A_ ≅ 0.1 materials would be too stiff, while below *ϕ*_A_ ≅ 0.01 dilution or shortening of the A-block hinders network formation. The architectural control over adhesion through [*n*_sc_, *n*_g_, *n*_x_] is maintained, while the additional levers of *n*_bb_, *n*_A_, and *ϕ*_A_ are used to improve bulk firmness and cohesive strength (Figure 5b inset, Table S3).^35^ Even at higher modulus, *E*_0_ ≅ 130 kPa, brush HM-PSAs demonstrate viscoelastic debonding at ε̇ = 1 s^–1^, where both tack and fibrillation are witnessed, suggesting their potential implementation as high shear adhesives (Figure 5b, Figure S18). This contrasts with brush elastomers, where greater stiffness results in a Rouse time shift constituting elastic debonding at the same strain rate. Lastly, characteristic of HM-PSAs, A-*g*-B network disassembly to a polymer melt at a relatively low temperature of ∼126 °C (Figure 5c) enables molding and additive manufacturing of biomedical devices (Figure 5d).

## Conclusion

In conclusion, we have developed a PSA design platform utilizing additive-free brush elastomers that empowers control over adhesive properties by encoding material relaxation. This architectural blueprint enables the programming of *W*_adh_ and debonding mechanisms by varying side chain length, grafting density, and length of the network strand in brush networks. Unveiling the fundamental structure-properties correlations between brush architecture and adhesive performance is a pivotal step toward universal design of PSAs. We plan to use this strategy to introduce previously unworkable chemistries into PSA materials and further study the effect of large strain-stiffening in brush HMPSAs.

## Supporting Information Available

Synthesis and molecular characterization of bottlebrush networks; mechanical properties and Rouse time measurements; theoretical analysis of the Rouse time of bottlebrush networks (PDF)
Movie S1. Probe tack tests of PBA brush PSAs with different side chain DP denoted by [*n*_sc_, *n*_g_]. Samples with increased side chain length resulted in greater work of adhesion and fibril elongation. This is a video representation in coordination with viscoelasticity shown in Figure 3a. MP4-1)
Movie S2. Hanging weight tests of PIB brush PSAs with different grafting density denoted by [*n*_sc_, *n*_g_]. Samples with increased grafting density resulted in being able to uphold larger loads. This is a video representation of Figure 3c where the hanging weight apparatus is 12 lb/in^2^ itself. (MP4-2)
Movie S3. PBA and PIB brush PSAs with the same modulus (*E*_0_∼ 30 kPa) and strain-stiffening (β ∼ 0.08) during probe tack testing. This acts as a visual representation of the quantitative observations denoted in Figure 4a. The PIB brush PSA sample has greater work of adhesion and tack. (MP4-3)
Movie S4. PIB and PBA brush PSA samples with different equilibrium mechanical properties but the same work of adhesion from strain rates 0.001 to 1s^–1^. This video is a visual representation of data in Figure S36 at ε̇ ∼ 1s^–1^. (MP4-4)
Movie S5. PBA brush PSAs with the same modulus (*E*_0_∼ 20 kPa) but different strain stiffening (β) debonding from a large probe. This acts as a visual representation of the quantitative observations denoted in Figure 4b. The larger β sample detaches before the sample with lower β. Samples were deboned at ε̇ ∼ 1s^–1^. (MP4-5)
