# Supplementary material for: Sticky Architecture: Encoding Pressure Sensitive Adhesion in Polymer Networks
Source: ACS Cent Sci. 2023 Feb 1;9(2):197–205. doi: 10.1021/acscentsci.2c01407 (PMC9951292; doi:10.1021/acscentsci.2c01407)
Supplement: Supplementary file 1 — oc2c01407_si_001.pdf [file oc2c01407_si_001.pdf]

Supporting Information

Sticky architecture: Encoding pressure sensitive adhesion in polymer networks

Mitchell Maw[1], Erfan Dashtimoghadam[1], Andrew N. Keith[1], Benjamin J. Morgan[1], Alexander K. Tanas[1], Evgeniia Nikitina[2], Dimitri A. Ivanov[2,3], Mohammad Vatankhah-Varnosfaderani[1], Andrey V. Dobrynin[1]*, Sergei S. Sheiko[1]*

[1]*Department of Chemistry, University of North Carolina at Chapel Hill, NC 27599-3290, USA*

[2]*Lomonosov Moscow State University; Leninskie Gory 1, 119991, Moscow, Russian Federation*

[3]*Institut de Sciences des Matériaux de Mulhouse-IS2M; CNRS UMR 7361, 15, rue Jean Starcky, F-68057 Mulhouse, France*

Correspondence to:
avd@email.unc.edu
sergei@email.unc.edu

1. Materials and Methods

Materials. Hexanes, tetrahydrofuran (THF), toluene, methanol, and N,N dimethylacrylamide were purchased from Fischer Scientific and used as received. Methyl-vinylidene terminated polyisobutylene (RB HR-PIB) oligomers with average molar mass of 1000, 1300, and 2300 (Đ ~ 1.9) were obtained from RB products and used as received. N-butyl acrylate (nBA, 99%) was obtained from Sigma Aldrich and purified via basic alumina column to remove inhibitor. In addition, α,ω methacryloxypropyl-terminated poly(dimethylsiloxane) (DMS-R18, average molar mass of 5000g/mol, Đ = 1.15) was obtained from Gelest and purified using basic alumina columns to remove inhibitor. Hydrogen bromide (HBr, 33% in ethyl acetate), tetrabutylammonium bromide (TBAB, 98%), phenylbis(2.4.6-trimethyl-benzoyl)phosphine oxide (BAPO), ethyl α-boromoisobutyrate (EBiB, 98%), ethylene bis(2-bromoisobutyrate) (2-BiB, 97%), methacrylic acid (99%), potassium tert-butoxide (potassium t-butoxide, 98%), tetrabutylammonium bromide (TBAB, 98%), basic alumina, neutral silica, and copper (I) bromide (Cu(I)Br) were obtained from Sigma Aldrich and used as received. Synthesis of potassium methacrylate (KOMA) was reported previously.[1] Linear polyisobutylene (average M_n~ 600,000 g/mol by GPC) was purchased from Sigma Aldrich and recast in toluene to make a film.

Gel-permeation chromatography (GPC). The molecular weight of the A-g-B brush copolymers synthesized by grafting through free-radical polymerization was determined by GPC using a Tosoh EcoSEC *Elite* GPC system equipped with a TSKgel Super HM-M (17392) column maintained at 40°C with an RI detector and Tosoh LENS^{TM} 3 multiangle light scattering detector. Tetrahydrofuran was used as the mobile phase at a flow rate of 0.5 mL/min. The molecular weight and dispersity was reported based on polystyrene standards. Note that molecular weight readings are reported from light scattering detection.

Static and Dynamic Mechanics of Brush elastomer PSA's. Both elastic and viscoelastic mechanical properties (Rouse time) were determined through uniaxial tensile testing. The E_0 of each brush PSA elastomer was determined by fitting the equation of state,

$$\sigma_{xx}(\lambda) = \frac{E}{9}(\lambda^2 - \lambda^{-1})\left(1 + 2\left[1 - \frac{\beta}{3}(\lambda^2 + 2\lambda^{-1})\right]^{-2}\right) \qquad \text{(S1)}$$

to the experimental stress-strain curve at a strain rate ($\dot{\varepsilon}$) along the elastic plateau. The output structural modulus, E, and β were subsequently used to calculate E_0 from the derivative of Eq. S1 as $\lambda \to 1$,[2]

$$E_0 = \frac{E}{3}(1 + 2(1 - \beta)^{-2}). \qquad \text{(S2)}$$

See example in Figure S15.

Uniaxial tensile testing. Uniaxial tensile testing was preformed using the G2-RSA Dynamic Mechanical Analyzer (TA Instruments) at a strain rate of 0.0001-0.005 s^{-1} depending on the sample (where elastic plateau began) at 20 °C. A dog-bone shaped sample was cut with dimensions of 12

mm × 2 mm × 1mm (exact thickness noted typically corresponding to h during probe test). Samples were stretched until catastrophic failure was reached.

Rheological data and the Chang viscoelastic window. An oscillatory frequency sweep at 5% strain in the range of the Chang viscoelastic window ($10^{-2}\ Hz - 10^{2}\ Hz$) was performed for all samples and distinct trends were observed with independent control of the elastomer structural code (ARES-G2 rheometer, TA instruments). The G' and G'' at the boundaries of the sweep were plotted at 20 °C and a Chang viscoelastic window was formed for each subset of PBA and PIB brush elastomer PSAs.

Rouse time determination of PIB and PBA brush PSA's. The Youngs modulus of the brush PSAs at small deformations, E_0, decays with time as,

$$E_0 \approx \begin{cases} 3\rho k_b T\left(\frac{\tau_0}{t}\right)^{\frac{1}{2}}, & for \quad \tau_0 < t < \tau_R \\ \frac{3\rho k_b T}{N}, & for \quad t > \tau_R \end{cases} \tag{S3}$$

supporting time independent relaxation above τ_R.[3] Utilizing the Boltzmann superposition principle, stress evolution at small deformations during tensile testing of the PSA is

$$\sigma_{xx}(t) \approx \dot{\varepsilon}\int_0^t E_0(\Delta t)d\Delta t \tag{S4}$$

at a constant $\dot{\varepsilon}$.[4,5] Stress relaxation for the networks follow stepwise time dependence of rate normalized stress,[3]

$$\frac{\sigma_{xx}(t)}{\dot{\varepsilon}} \approx E_0\tau_R^{1/2}t^{1/2} \sim t^{1/2}, \qquad for \quad t < \tau_R \tag{S5}$$

and,

$$\frac{\sigma_{xx}(t)}{\dot{\varepsilon}} \approx E_0 t \sim t, \qquad for \quad t > \tau_R. \tag{S6}$$

Work of adhesion (W_{adh}) measurements. The W_{adh} is measured using a modified version of the probe tack test using a G2-RSA DMA.[6] The top arm contained a 2 mm diameter probe and the bottom a 25 mm plate with roughness of 0.5 microns (TA instruments). Segments of sample were placed on the bottom compression plate and allowed to wet the surface over time. In addition, a rubber roller was used to apply light pressure to ensure the adhesive bond between the elastomer and bottom plate (acting as carrier) remained intact during measurement. The run consisted of compression at 0.01 mm/s until a contact pressure, $P = 1\ MPa$, was attained. The probe was held at a dwell time, $t = 100\ s$ and removed at 1mm/s for debonding.

Tensile hanging weight test. The hanging apparatus contained a curved steel loop attached to a level steel plane. A hook attached to a bottom pan was used to hold 10g weights and the hook was linked to the aforementioned steel bar. A 2mm sample was prepared by wiping down the surface with acetone, allowing any solvent to evaporate, and forming the adhesive bond by wetting one side of the adhesive to a steel horizontal wall and the other side of the PSA apparatus via the level steel plane. The sample was allowed to wet the surface for 100 seconds under pressure. The

apparatus without any weight was inverted so the pan was hanging and being upheld by the adhesive alone. The 10g weights were added sequentially until failure of the adhesive bond. The Tensile hanging stress was determined at the point of last weight addition.

Fused filament fabrication 3D printing. Fused-filament fabrication 3D printing was performed with a poly[nBA-*ran*-MMA-*g*-(PIB/PS)] thermoplastic elastomer sample using a Cellink BioX 3D printer where shape stl. files were created with Tinkercad in the shapes of biomedical adhesives (Figure 5d). The polymer reservoir was heated to 150°C to ensure adequate flow and extruded at a pressure of 120 kPa.

Structural verification of brush structure by small-angle X-ray scattering (SAXS).[1] The SAXS measurements were carried out at the ID02 beamline of the European Synchrotron Radiation Facility (ESRF) in Grenoble, France. The experiments were conducted in transmission geometry using a photon energy of 12.46 keV. The recorded 2D data were centered, calibrated, regrouped and reduced to 1D using the SAXS utilities platform described elsewhere.[7] The analysis of the SAXS data was performed using the SANS data reduction and analysis package provided by NIST[8] for the Igor Pro environment (WaveMetrics Ltd.).

The monochromatic incident X-ray beam was collimated on the sample to a footprint of 100×200 μm^2 (V×H). The total photon flux was estimated to be 9.10^{11} ph/s allowing for acquisition times of less than 100 ms. The accessed q values, with $|q| = 4\pi.\sin(\Theta)/\lambda$, where Θ is the Bragg angle and λ – wavelength, cover a range from 7.0×10^{-3} nm^{-1} to 5.0 nm^{-1}. A Rayonix MX-170HS implemented in a 35m long vacuum flight tube was applied for recording of SAXS intensities at two different sample-to-detector distances of 1.5 and 10.0 m, respectively. For optimization of the scattering signal, a binning of 2×2 pixels was applied resulting in an effective pixel size of 89µm in both directions.

2. Synthesis and Characterization

Anti-markovnikov bromination of HR-PIB 1000. A 250 mL round bottom flask was prepared with a stir bar and 50 g (0.05 mmol) RB HR-PIB ($M_n = 1000 g/mol,\ Đ \sim 1.9$) dissolved in hexane (150 mL) and placed in an ice bath. The solution was bubbled with air for 30 minutes at 0°C and 24.3 g of 33 w/w% HBr (0.1 mol) in EtOAc was added dropwise to the flask with vigorous stirring. The solution reacted for 2 hrs at 0°C followed by RT o/n. Stirring was ceased and the resultant anti-Markovnikov bromine functionalized PIB oligomer was washed with H_2O/Na_2CO_3 twice (dried with anhydrous $MgSO_4$) and extracted with a SiO_2 column. The hexanes were evaporated by bubbling with air yielding 88% functionalized polymer (determined by H^1-NMR, Figures S2-4). No residual olefin residue was present suggesting higher yield.

Synthesis of PIB ($n_{sc} = 18$) macromonomer. The functionalized oligomer was dissolved in THF (100 mL) and transferred to a clean 250 mL round bottom flask equipped with a stir bar. The solution was charged with 18.6 g KOMA (0.15 mol) and 48.3 g TBAB (0.15 mol) and ran 24 hrs at 45°C. The solution was centrifuged to remove residual salt and unreacted reagent. Subsequently, the solution was condensed by bubbling with air and washed with H_2O/hexane twice. The organic layer was separated and ran through a SiO_2 column revealing PIB ($n_{sc} = 18$) macromonomer product (96% yield). Again, no residual peaks were present from the α-hydrogens suggesting higher yield. ^{1}H-NMR of the synthetic progression is shown in Figures S2-4. This synthesis applies to RB HR PIB-1300 ($n_{sc} = 23$) and RB HR PIB-2300 ($n_{sc} = 41$) with molar ratios respectively applied.

Synthesis of butyl acrylate by SARA ATRP. Poly(*n*-butyl acrylate) with different degree of polymerization were synthesized by supplemental activation reducing agent (SARA) atom transfer radical polymerization (ATRP) followed by a post polymerization functionalization displacing the bromine end group with potassium methacrylate. To a 500 mL air free Schlenk flask 120 g (0.94 mol) of butyl acrylate was combined with Me_6TREN (10 μL, 37 μmol), $CuBr_2$ (8 mg, 36 μmol), and EBiB, 15.2, 7.3, or 3.7 g (0.078, 0.037, or 0.019 mol) depending on desired n_{sc}, and diluted with an equal volume of acetonitrile. The reaction mixture was then cooled with an ice bath and oxygen was removed by bubbling nitrogen gas for 1 hour. The polymerization was initiated by adding a stir bar equipped with a clean Cu^0 wire and transferring to 45 C mineral oil bath. The reaction was monitored by ^{1}H NMR and stopped near 80% conversion with the addition of chloroform. Excess catalyst was removed by washing in water ~11 times and excess solvent was removed by rotary evaporation at 45 C under reduced pressure.

Synthesis of poly(*n*-butyl acrylate) macromonomers. The previously synthesized poly(*n*-butyl acrylate) was dissolved in 7 parts *N*,*N*-dimethylacetamide. Potassium methacrylate was added in large excess (>3 molar equivalents) and the reaction was left to stir for 3 days and turning a faint yellow color. To purify, the mixture was 1 part chloroform and 1 part water were added separating the mixture into two separate phases. The aqueous phase was discarded and the remaining organic

component was an addition 11 times with water until clear. Solvent was removed by rotary evaporation.

Synthesis of poly(n-butyl acrylate) macro-crosslinker by SARA ATRP. Poly(n-butyl acrylate) macro-crosslinkers were synthesized using an equivalent procedure to the poly(n-butyl acrylate) macromonomers. The one exception is that a difunctional 2-BiB ATRP initiator was used to polymerize n-BA such that the corresponding macromonomer was functionalized at both ends of the polymer chain. This difunctional poly(butyl acrylate) macro-crosslinker was also synthesized on a much smaller scale due to relatively small amount of it used during synthesis. The $n_{sc} = 80$ crosslinker was synthesized by combining 24 g (0.19 mol) of butyl acrylate, Me_6TREN (2 μL, 7.4 μmol), $CuBr_2$ (1.6 mg, 7.2 μmol), and 2-BiB (0.67 g, 1.9 μmol) and diluting the mixture to 50% with acetonitrile. The reaction was then cooled in an ice bath and degassed for 1 hour with bubbling nitrogen gas. The polymerization was initiated by the addition of a Cu^0 wire and transferred to a 45 C oil bath until the reaction reached ~80% conversion. The reaction was then terminated by the addition of 50 mL of chloroform and washed 11 times in water. Solvent was removed by rotary evaporation at 45 C under reduced pressure. The cleaned polymer was then functionalized by the addition of 7 parts N,N-dimethylacetamide and a large excess of potassium methacrylate and left stirring for 72 hours. 50 mL of chloroform and 100 mL of water were then added separating the polymer into the organic phase. The organic phase was then washed in water 11 times until it became clear. Solvent was again removed by rotary evaporation at 45 C under reduced pressure.

Synthesis of PIB brush elastomer PSAs (Scheme 1). A scintillation vial was charged with 5 g of PIB ($n_{sc} = 18$) macromonomer (5 mmol), THF (5 mL), and R18 according to n_x (ex. for $n_g = 1, n_x = 100$, R18 = 0.125 g). The vial was covered in aluminum foil and placed in an ice bath to prevent auto-initiation of R18. Furthermore, 7.0 mg (0.14 mol%) of BAPO was added to the vial. The vial was rapidly fixed with a rubber septum and was bubbled with nitrogen for 30 min. The deoxygenized solution was injected into a nitrogen flushed, hand-made glass mold and set to cure in a nitrogen chamber o/n (18-24hrs). The polymers were removed from the mold, swollen in THF twice to remove unreacted macromonomer (gel fraction > 90%). The PIB bottlebrush elastomers was dried o/n in fume hood followed by 2 hrs in in the oven at 60℃.

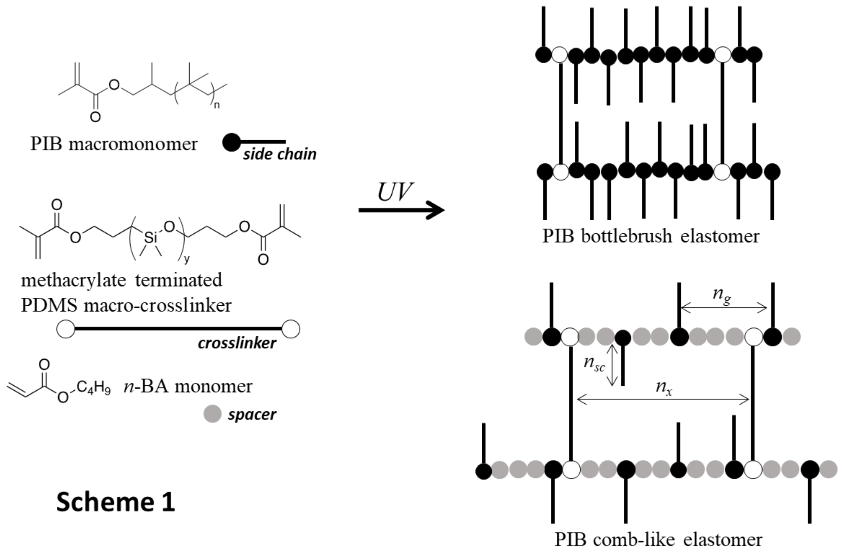

Scheme 1

Synthesis of bottlebrush poly(*n*-butyl acrylate) elastomers (Scheme 2). Bottlebrush poly(*n*-butyl acrylate) elastomers were synthesized by combining macromonomer (4 g), macro-crosslinker (1, 0.5, and 0.25 mol%), and BAPO (1.5 wt.%) were diluted to 50% in anisole. Nitrogen gas was used to purged with oxygen for 1 hour and then the mixture was injected into 1.3 mm thick elastomer molds and left to polymerize overnight in nitrogen atmosphere. The corresponding film was separated from its mold and a small portion was set aside to measure the samples corresponding gel fraction. The larger bulk part of the film was washed 3 times in toluene and dried prior to measurement. Gel fractions were for the most part at or above 90%. Gel fractions were measured by washing small sections of unwashed films in toluene 3 times over the course of 72 hours. The mass post washing divided by the mass of the gel fraction after washing was taken to be the gel fraction.

Synthesis of poly(butyl acrylate) comb elastomers (Scheme 2). Poly(butyl acrylate) comb elastomers were synthesized by combining macromonomer, crosslinker (1, 0.5, and 0.25 mol%), BAPO (5-10 mg), and *n*-BA as spacer. The mixture was purged of oxygen using bubbling nitrogen gas and injected into a 1.3 mm molds and left to polymerize under ambient light conditions under a nitrogen atmosphere. As a specific example, for an [11,10,100] sample, $[n_{sc}, n_g, n_x]$ 4 g of $n_{sc} = 11$ macromonomer, 3g n-BA spacer (9 molar equivalents), 0.0339 g (0.05 molar equivalents), and 5 mg of BAPO were used. A small portion of the film was removed to measure the gel fraction and the bulk part of the elastomer was washed 3 times in toluene and dried prior to sample measurement.

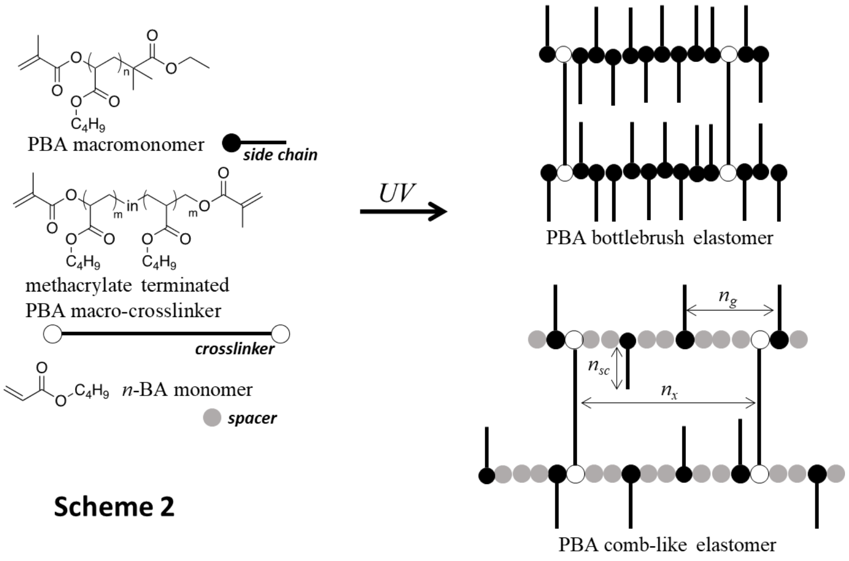

Scheme 2

A-g-B brush copolymers (HMPSAs)[9]

Synthesis of PS oligomers. ATRP of polystyrene homopolymer was performed with a target n_A = 60 at 34% conversion to avoid large viscosities at higher conversion. Styrene (100g, 0.96mol), HEBIB (1.16g, 5.5mmol), PMDETA (0.095g, 0.114mL, 0.55mmol), and a stir bar were added to a Schlenk flask. The solution was bubbled with dry nitrogen for 1 hour then Cu(I)Br (0.079g, 0.55mmol) was quickly added to the reaction mixture under nitrogen atmosphere. The flask was sealed, purged for an additional 15 minutes, and then immersed in an oil bath at 90°C. The reaction mixture was left to polymerize for 14 hrs to receive a 34% conversion (n_{sc} = 60) and the reaction was quenched by exposing the mixture to oxygen (Figures S8). The mixture was centrifuged and gravity filtered to remove residual Cu-ligand complex. Residual styrene monomer was evaporated and the remaining PS oligomer was dissolved in minimal THF and crashed in excess methanol (1:10, THF:Methanol by volume) 3 times. The washed PS oligomer was dried overnight at room temperature under reduced pressure to remove any residual solvent. The PS oligomer (30 g, 4.8mmol, DP = 60) was transferred to a round bottom flask sealed by rubber septum and parafilm and dissolved in 60 ml of THF. Once the PS was fully dissolved, 0.05g (47μL, 80μmol) dibutyltin dilaurate was added to the solution, it was subsequently purged of oxygen by bubbling the solution with nitrogen for 10 minutes. IEM (0.82g, 0.75mL, 5.3mmol) was added dropwise to the round bottom flask under constant stirring. Nitrogen was removed from the flask, and the solution was set to stir for 18hr. The subsequent solution was further diluted with THF (5-10x) and passed through silica column twice. The purified mixture was dried under reduced pressure and characterized by ^{1}H-NMR (Figure S8). H^1-NMR reveals 80% conversion so subsequent calculations for ϕ_A for performed considering an 80% ratio of macromonomers.

FR polymerization of A-g-B brush copolymers by grafting through. A Schlenk flask was charged with appropriate molar quantities of side-chain macromonomer (PDMS, PIB), spacer (n-BA), A-block macromonomer (PS), 1:1 volume of p-xylene, and 0.15 mol% initiator (BAPO). The

flask was shielded from light and purged with nitrogen for 30 minutes. Subsequently, the solution was removed from nitrogen and allowed to polymerize under UV-light for 18hr. The solution exhibits a light-yellow color upon introduction to the UV-light but returns to transparent after polymerization. The unwashed polymer solution was caste in a Teflon mold at 60°C and dried overnight. The resultant polymer was washed according to chemistry. poly[nBA-*ran*-MMA-*g*-(PIB/PS)]: the unwashed polymer was dissolved in THF and crashed with acetonitrile (1:1.2, THF:acetonitrile) 3 times. The washed polymer was dissolved in p-xylene once more and caste into a Teflon petri-dishes (Welch Fluorocarbon) at 60°C and dried overnight to be characterized by ^{1}H-NMR where no macromonomer peaks remained (Figure S9). Note that unreacted reagents act as diluent and decrease the modulus of the network. Comparing washed and unwashed A-g-B network stress-strain curves exhibit this behavior. The number average molecular weight (M_n) of the A-g-B brush polymer stand was determined by light scattering detection during gel-permeation chromatography (Figure S10).

Ex. synthetic calculations. poly[nBA-*ran*-MMA-*g*-(PIB/PS)]. Sample: 030722_2. 2.00g n-butyl acrylate (15.6mmol), 2.23g PIB macromonomer (2.23mmol), 0.46g PS macromonomer (16.5μmol, 80% conversion PS mixture, total mass 0.56g), 7mg BAPO (17μmol, 0.15w/w%), and 4.5mL of p-xylene.

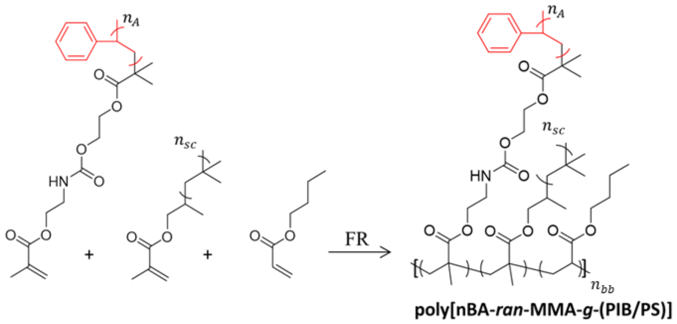

Scheme 3. Polymerization of poly[nBA-*ran*-MMA-*g*-(PIB/PS)] brush graft copolymers.

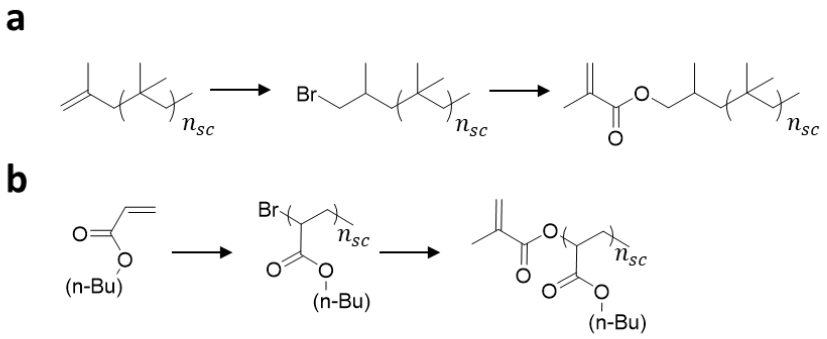

Figure S1. Synthesis of a) PIB macromonomer (Figures S2-4) and b) PBA macromonomer for brush elastomer PSAs (Figures S5-7).

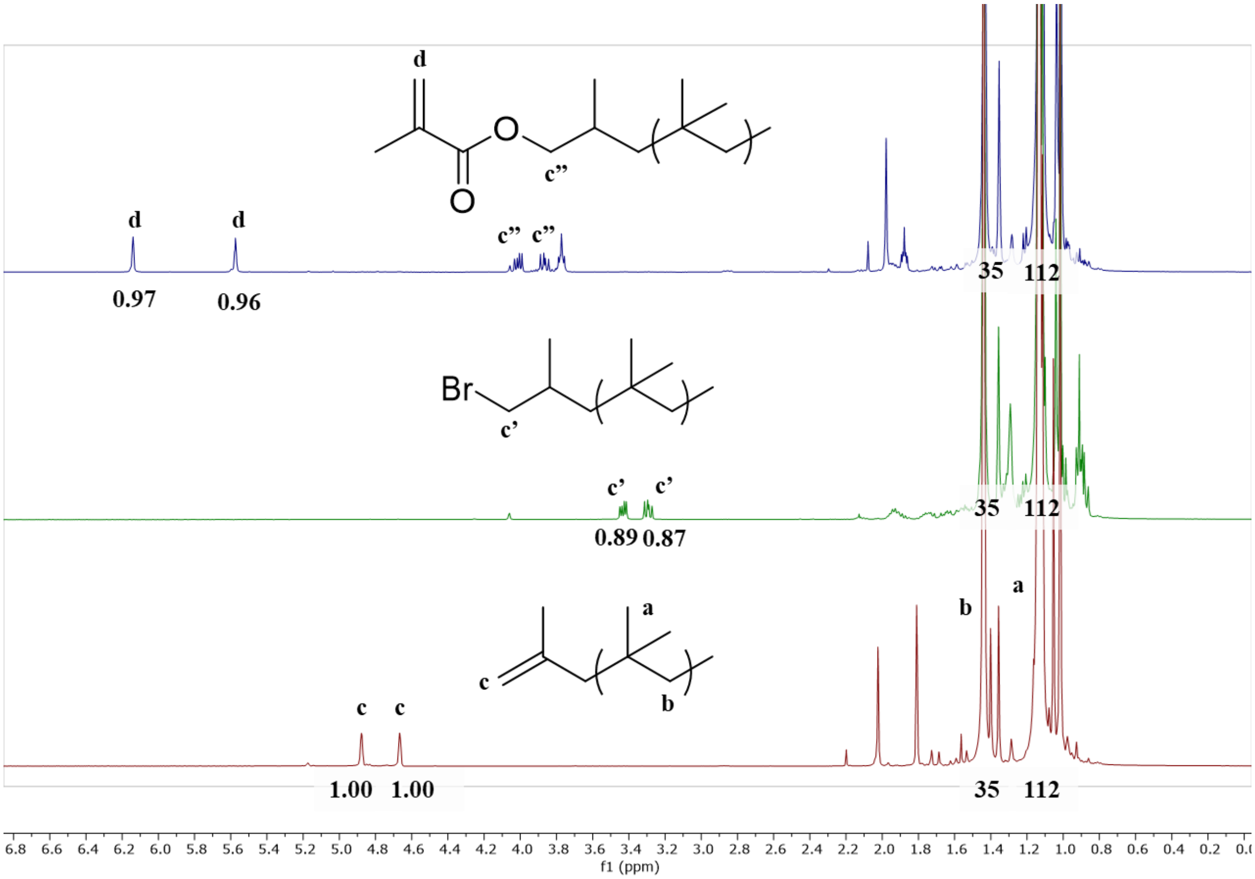

Figure S2. ^{1}H-NMR of PIB ($n_{sc} = 18$) Macromonomer Synthesis. (400MHz, $CDCl_3$): 6.14, 5.58 (C**H₂**=C(CH_3)-C=O, PIB macromonomer, s, 1H), 4.67, 4.88 (C**H₂**=C-CH_3-, methylvinylidene PIB, s, 1H), 3.43, 3.30 (BR-C**H₂**-C-, brominated PIB, dd, 1H), 1.44 (-C**H₂**-C(CH_3)$_2$-, PIB, s, 35H) 1.15 (CH_2-C-(C**H₃**)$_2$-, PIB, s, 112H). PIB macromonomer was washed with H_2O and run through SiO_2 column twice to remove unfunctionalized PIB.

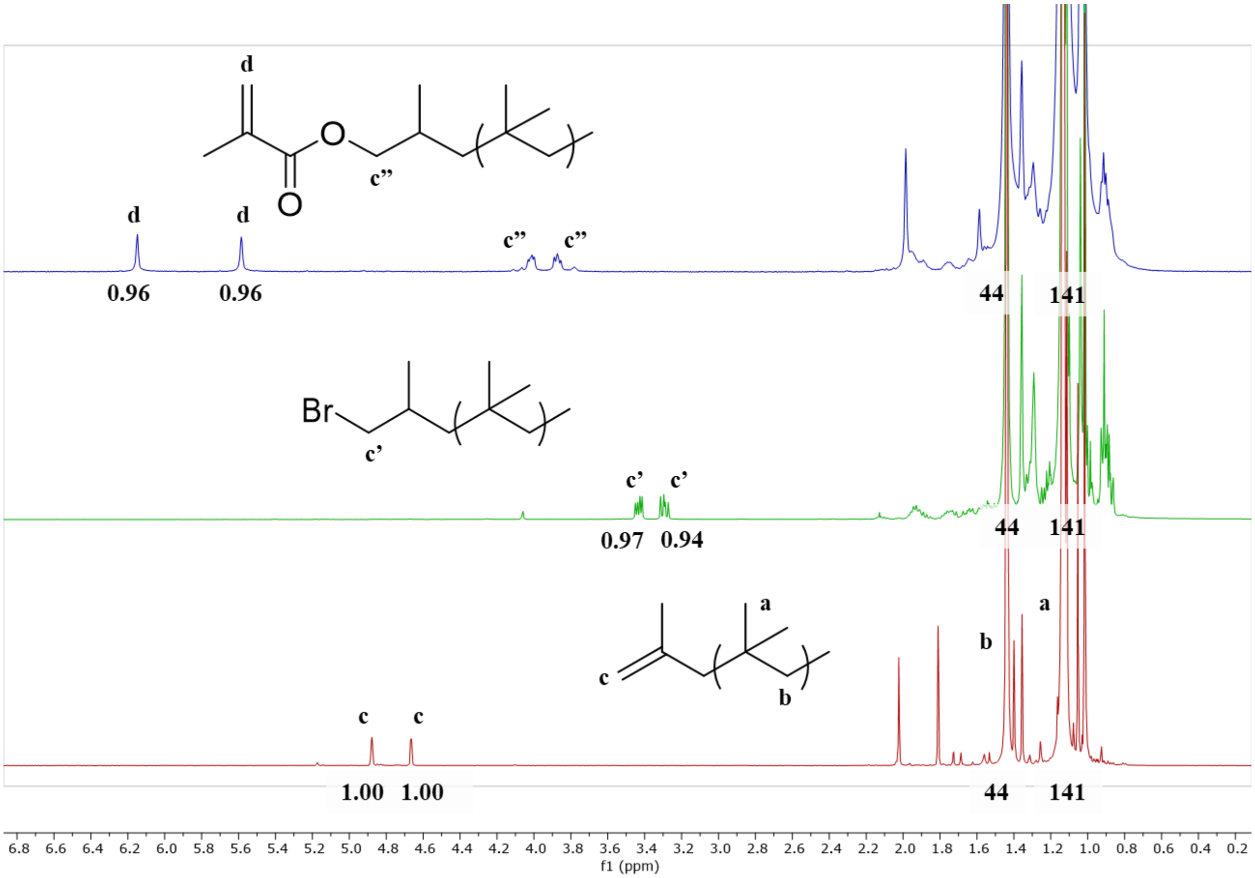

Figure S3. ^{1}H-NMR of PIB ($n_{sc} = 23$) Macromonomer Synthesis. (400MHz, $CDCl_3$): 6.14, 5.58 (C**H2**=C(CH_3)-C=O, PIB macromonomer, s, 1H), 4.67, 4.88 (C**H2**=C-CH_3-, methylvinylidene PIB, s, 1H), 3.43, 3.30 (BR-C**H2**-C-, brominated PIB, dd, 1H), 1.44 (-C**H2**-C(CH_3)2-, PIB, s, 44H), 1.15 (CH_2-C-(C**H3**)$_2$, PIB, s, 141H). PIB macromonomer was washed with H_2O and run through SiO_2 column twice to remove unfunctionalized PIB.

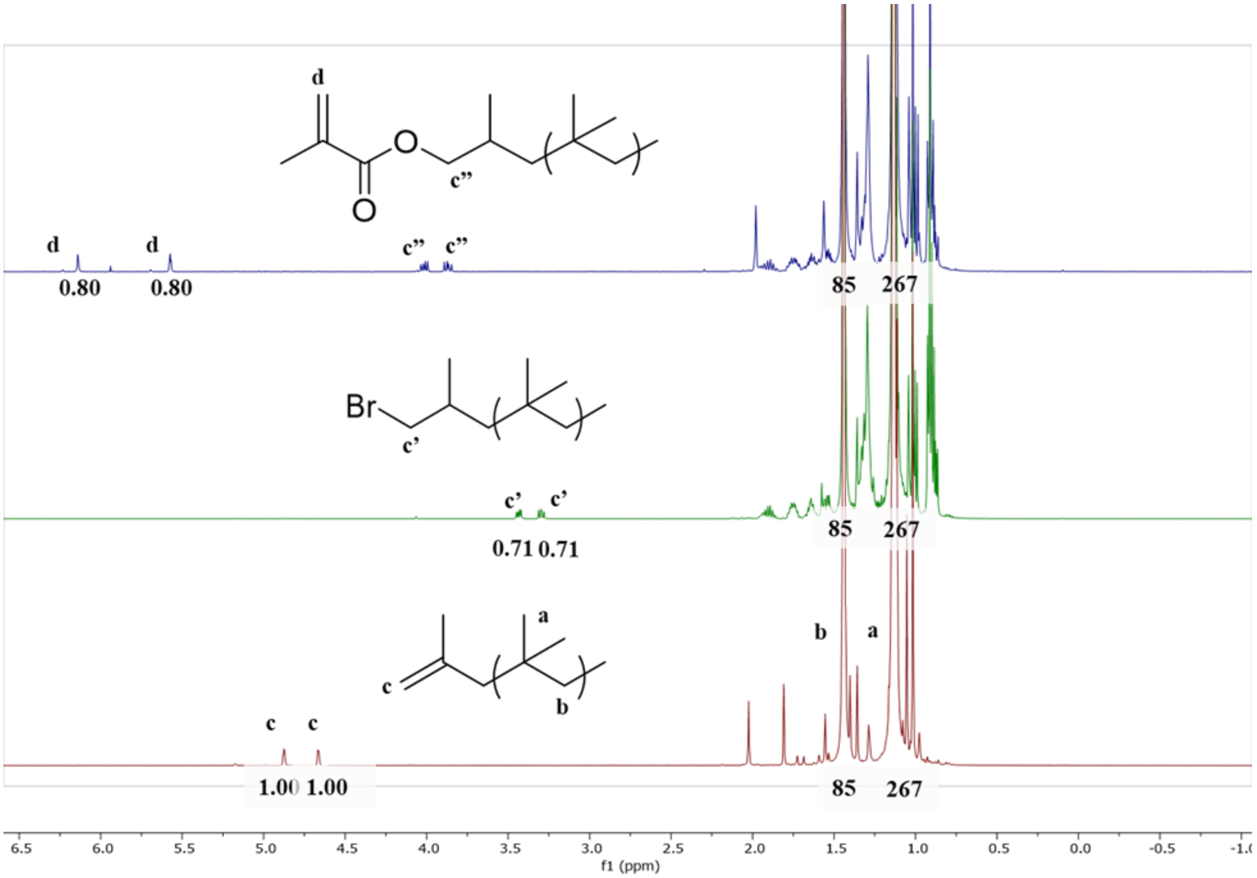

Figure S4. ^{1}H-NMR of PIB ($n_{sc} = 41$) Macromonomer Synthesis. (400MHz, $CDCl_3$): 6.14, 5.58 (C**H2**=C(CH_3)-C=O, PIB macromonomer, s, 1H), 4.67, 4.88 (C**H2**=C-CH_3-, methylvinylidene PIB, s, 1H), 3.43, 3.30 (BR-C**H2**-C-, brominated PIB, dd, 1H), 1.44 (-C**H2**-C(CH_3)2-, PIB, s, 82H), 1.15 (CH_2-C-(C**H3**)$_2$-, PIB, s, 246H). PIB macromonomer was washed with H_2O and run through SiO_2 column twice to remove unfunctionalized PIB.

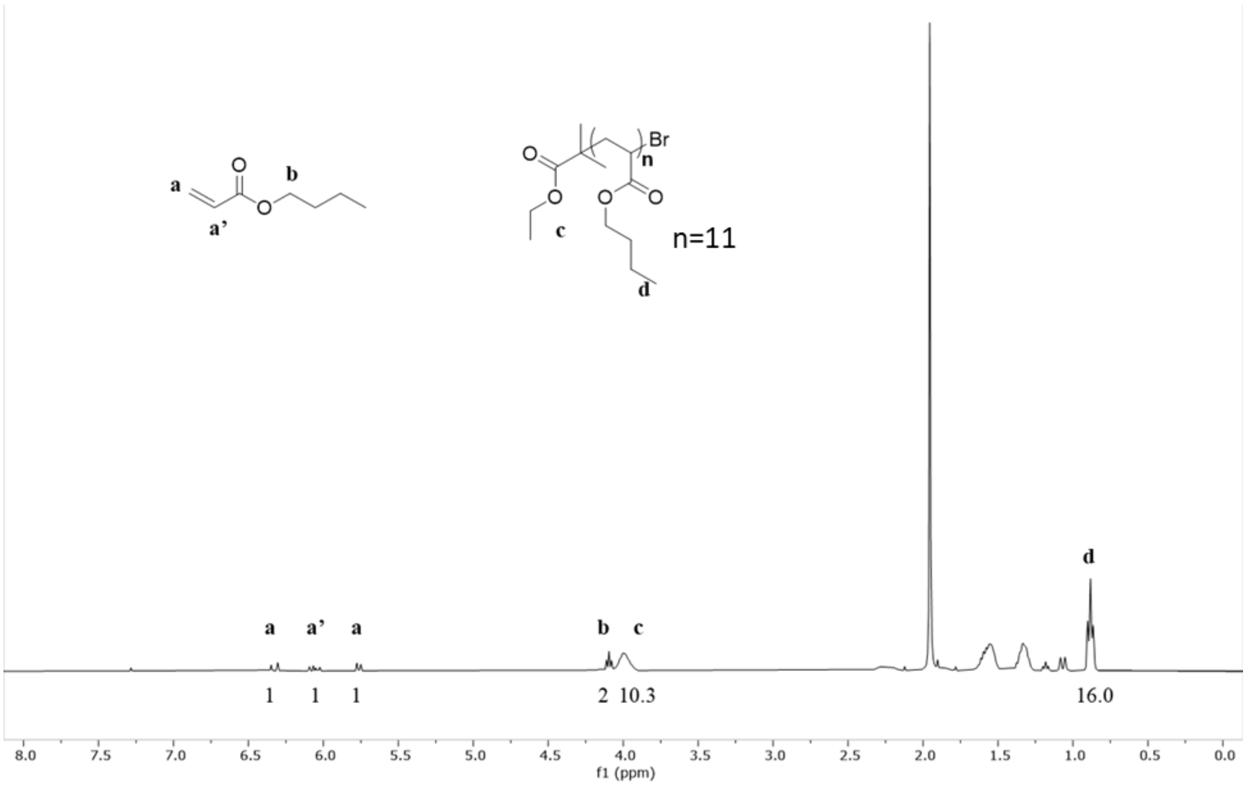

Figure S5. ^{1}H-NMR of unpurified *n*-butyl acrylate at completion of ATRP reaction. (400 MHz, $CDCl_3$): 6.3, 5.77 (C$\mathbf{H_2}$=C(H)C=O, d, 1H), 6.06 (CH_2=C($\mathbf{H}$)C=O, dd, 2H), 4.1 (-O-C$\mathbf{H_2}$-$(CH_2)_2$-CH_3, t, 2H) 4.00 (O-C$\mathbf{H_2}$-(CH_2)2-CH_3, s, 10.3H), 0.88 ((-O-CH_2-$(CH_2)_2$-C$\mathbf{H_3}$, t, 16H). The extent of reaction was taken to be the area ratio of peak $c/(b + c) = 0.84$.

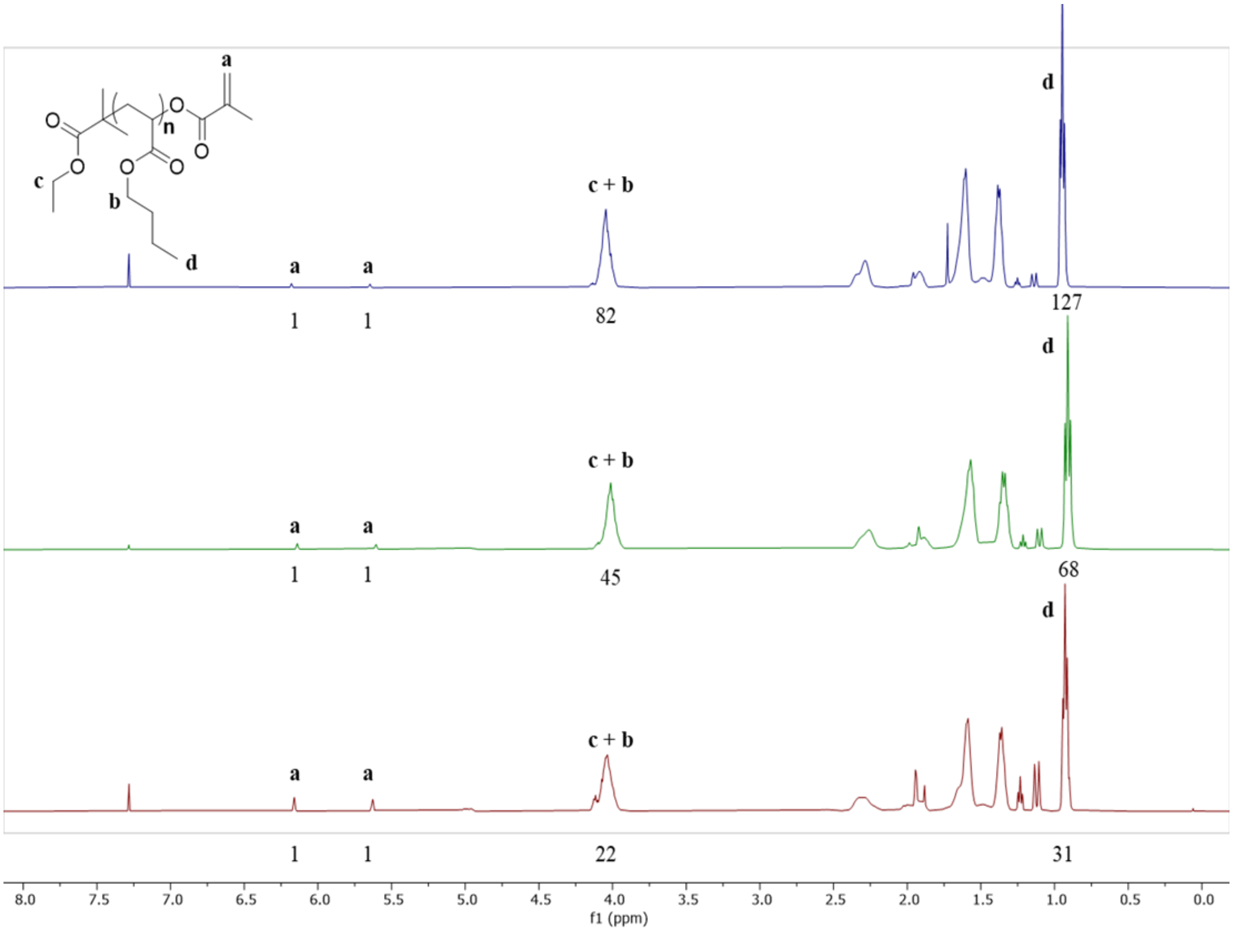

Figure S6. ^{1}H-NMR of functionalized poly(n-butyl acrylate) macromonomer. (400 MHz, $CDCl_3$): 6.14, 5.60 (C**H2**=C(CH_3)-C=O, s, 1H), 4.01 (-O-C**H2**-(CH_2)$_2$-CH_3, s, 22H), 0.88 (-O-CH_2-(CH_2)$_2$-C**H3**, t, 33H). The n_{sc} was taken as $(c + b)/2 \approx 11$.

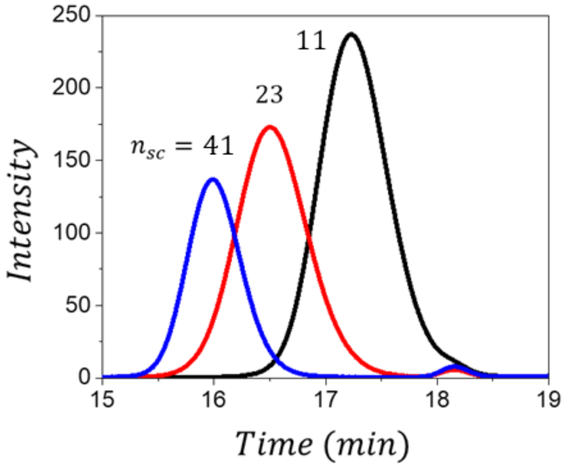

Figure S7. Gel permeation chromatographs of the synthesized poly(n-butyl acrylate) macromonomers. The dispersity of the corresponding macromonomers was found to be 1.12, 1.08, and 1.07 for the corresponding $n_g = 11, 23, 41$.

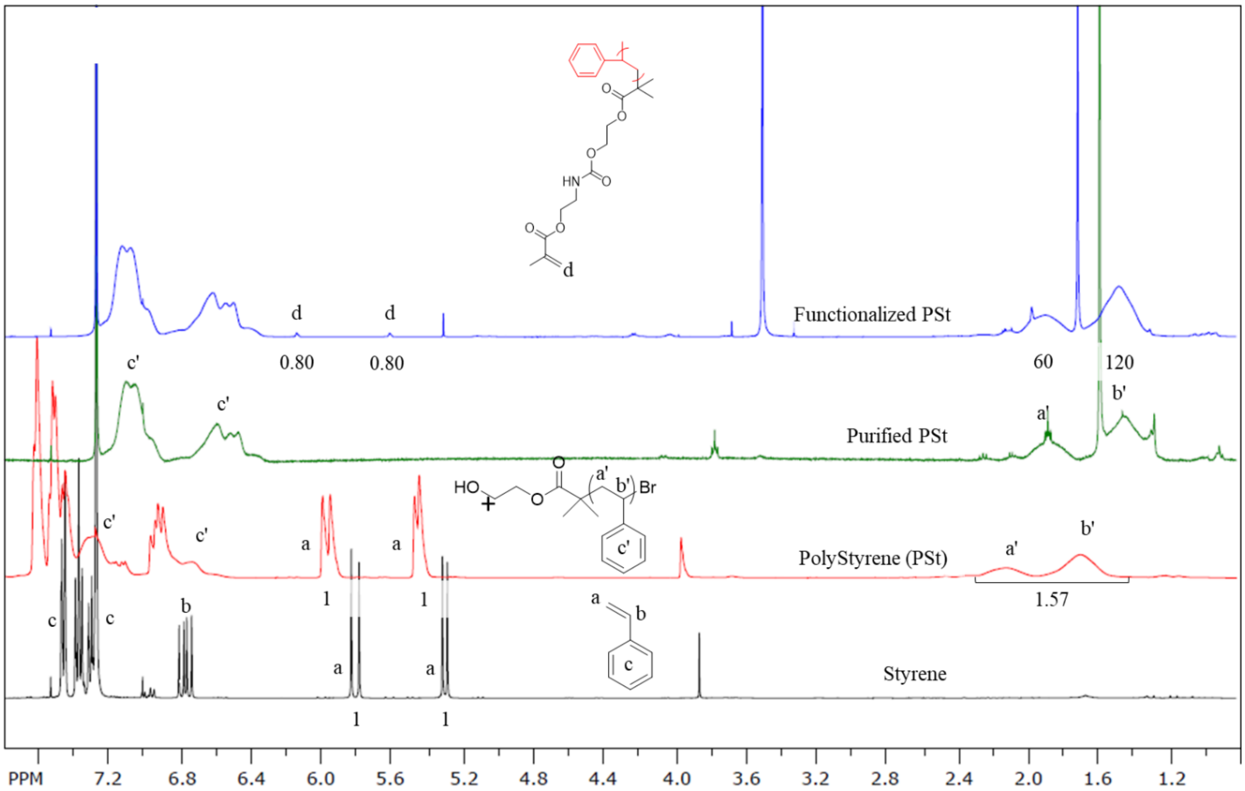

Figure S8. ^{1}H-NMR of functionalized polystyrene (PS) macromonomers used in FR polymerization at different stages (400 MHz, $CDCl_3$): 7.66-6.31 (C_6**H_6**-, residual styrene and PS, m, 6H), 6.15, 5.61 (C**H_2**=C(CH_3)-C-, functionalized PS, s, 1H), 5.70, 5.46 (C**H_2**=CH-, residual styrene, s, 1H), 2.16-1.27 (-C**H_2**-C**H**-, PS, m, 3H). 3.50, 1.71 (residual methanol), 1.58 (residual water). n_A = [styrene]/[I]*(a'+b')/3/[a/1+ a'+b')/3] = 175 * 1.57/3/[1+1.57/3] = 175*0.34 = 60.1.

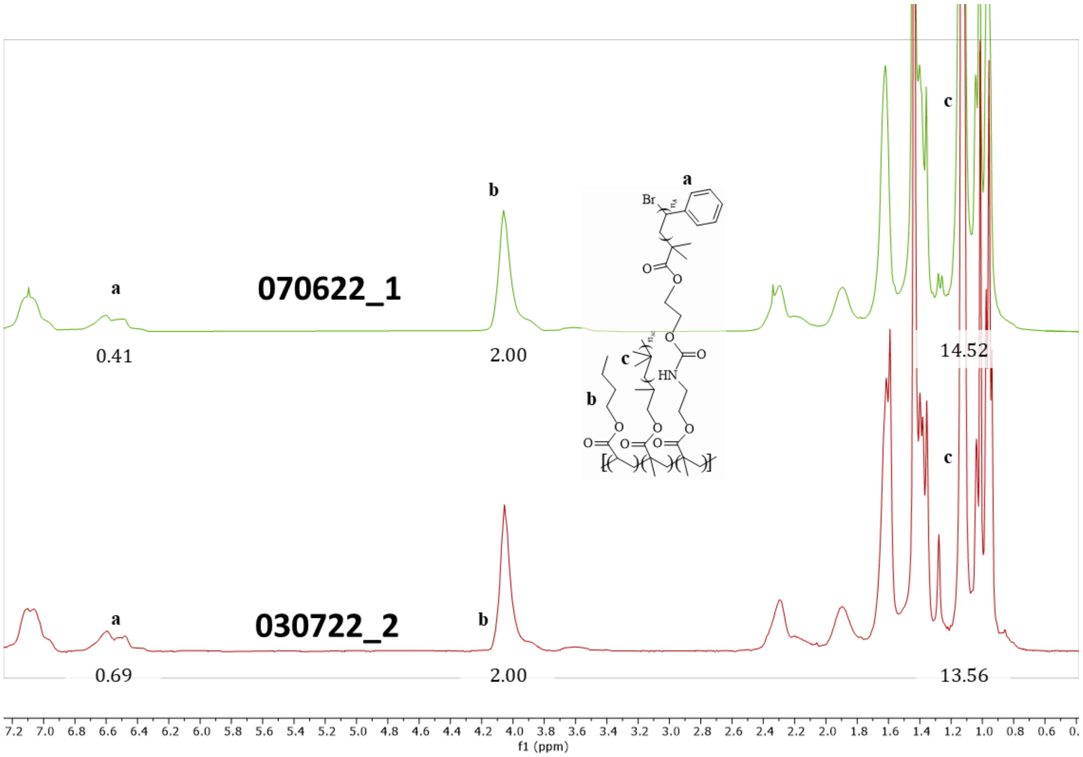

Figure S9. ^{1}H-NMR of purified poly[nBA-*ran*-MMA-*g*-(PIB/PS)] HMPSAs. (400 MHz, $CDCl_3$). 6.56 (CH-CH=C**H**-C-, PS side chain, d, 120H), 4.10 ((C=O)-O-C**H**$_2$-, PBA spacer, t, 2H), 1.15 (CH_2-C-(C**H**$_3$)$_2$-, PIB side chain, s, 112H).

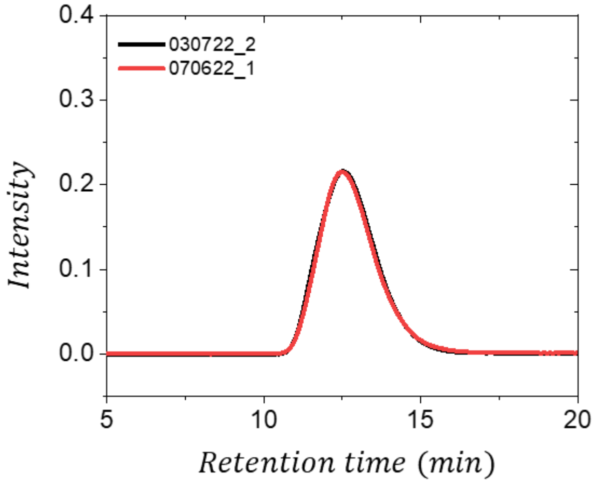

Sample	M_n (kg/mol)	Đ
030722_2	327	1.78
070622_1	305	2.09

Figure S10. GPC of poly[nBA-*ran*-MMA-*g*-(PIB/PS)] HMPSAs. 1mg/mL, HALS. Samples names reference to Table S3. The samples were run on a 30-minute cycle.

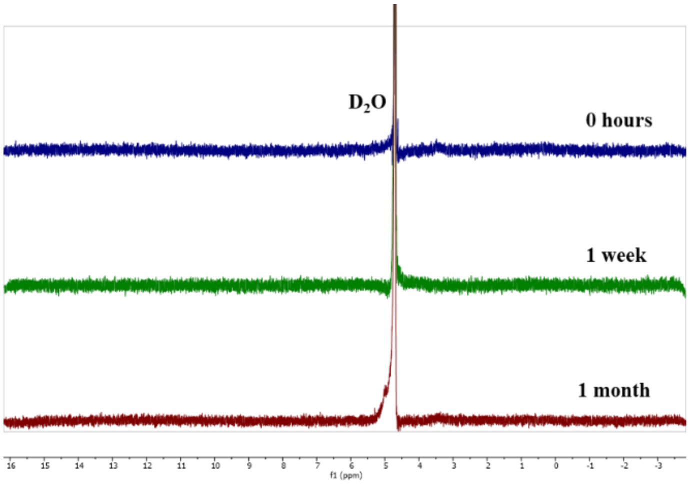

Figure S11. ^{1}H-NMR of PIB brush elastomer PSA [18,1,100] leachability (400MHz, D_2O): 4.75 (**D**$_2$O, solvent, 2D). Through aqueous leaching tests, ^{1}H-NMR reveals minimal small molecule drift from brush adhesive to solvent.

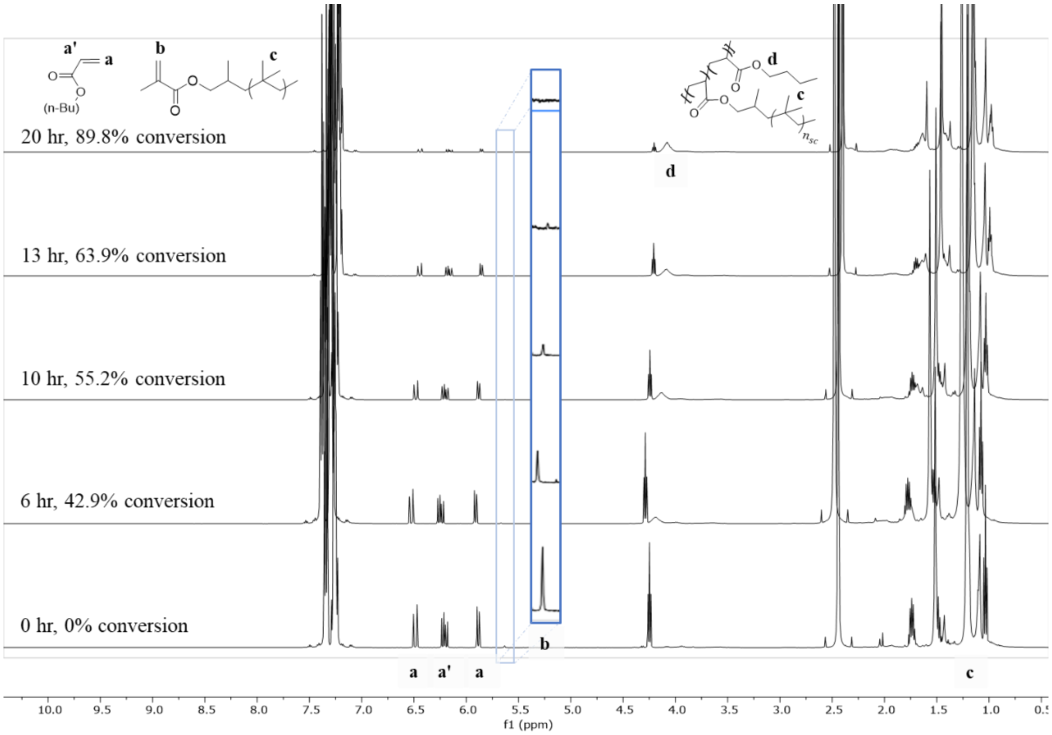

Figure S12. ^{1}H-NMR of copolymerization of PIB macromonomer and nBA. (400MHz, $CDCl_3$): 1.15 (CH_2-C-(CH_3)$_2$-, PIB, s, 112H), 5.60 (CH_2=C-CH_3-, PIB macromonomer, s, 1H), 6.20 (CH_2=CH-(C=O)$_2$-), nBA, q, 1H), 6.45, 5.88 (CH_2=CH-(C=O)$_2$-), nBA, d, 1H). nBA and PIB macromonomer were polymerized in accordance with $n_g = 4$ (4 mol:1 mol, respectively) by atom transfer radical polymerization (ATRP) to study the addition of monomer over 20 hours. The polymerization of brush copolymer was monitored by consumption of PIB macromonomer and nBA monomer. This is understood as an analog to our free-radical UV-curing methodology and concede potential effects of catalyst and ligand interactions in ATRP.

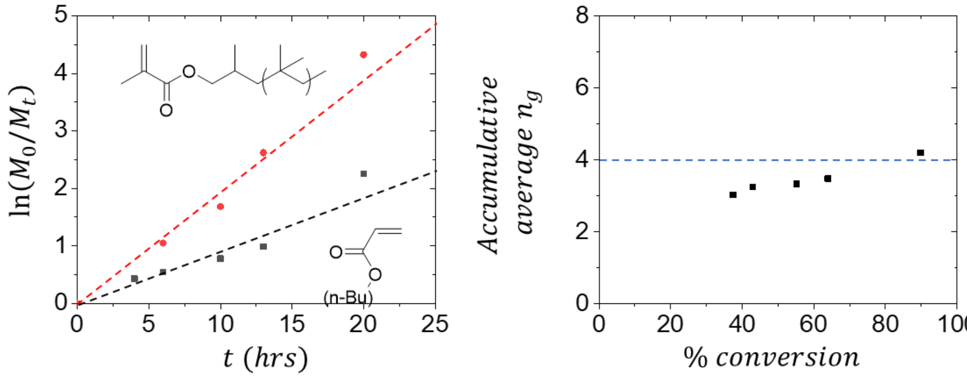

Figure S13. Copolymerization of PIB macromonomer and nBA. a) Monomer consumption with respect to time. PIB macromonomer ($n_{sc} = 18$) reacts at a faster rate than nBA monomer of the reaction. b) Accumulative n_g in agreement with monomer consumption. Greater reactivity of PIB macromonomer yields a gradient copolymer structure with greater PIB concentration on one end of the polymer strand and greater linear PBA concentration on the other.

4) SAXS Analysis

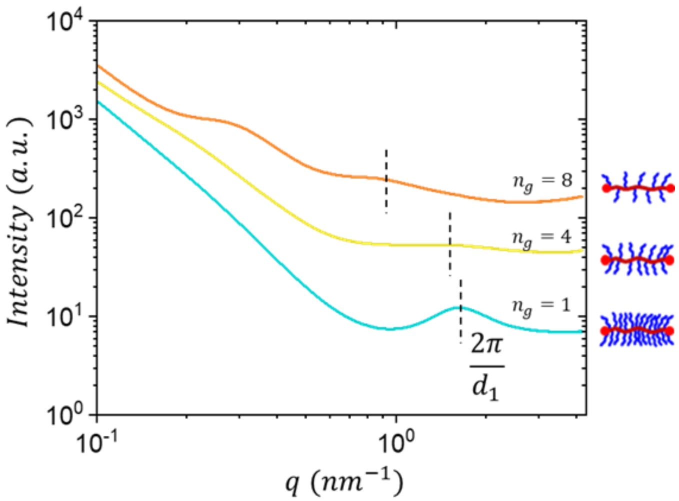

Figure S14. SAXS spectra of PIB elastomers from bottlebrush (SBB) to comb regimes. Densely grafted ($n_g = 1$) PIB elastomers exhibit a defined interbrush peak, d_1, while the peak broadens with larger n_g. At $n_g = 8$, a lower-q peak is ascribed to phase separation between the PIB side chains and PBA spacers in the backbone.

Table S2. Inter-brush distance for PIB bottlebrush and comb elastomers.

$[n_{sc}, n_g, n_x]$ (1)	d_1(nm) (2)
PIB Brush PSAs	
[18,1,100]	3.85
[18,4,100]	4.50
[18,8,100]	6.71

(1) Brush architectural code. (2) Inter-brush distance.

4. Mechanical properties

Table S1. Mechanical properties and Rouse time of brush elastomer PSAs.

$[n_{sc}, n_g, n_x]$ 1)	$E_0(kPa)$ 2)	β 3)	τ_R (s) 4)	regime5)
PIB Brush PSAs				
[18,1,100]	13.4	0.151	31.0	brush
[18,1,150]	6.75	0.089	77.5	brush
[18,1,200]	4.42	0.068	125	brush
[18,1,300]	3.32	0.046	510	brush
[18,2,100]	13.5	0.100	7.40	brush
[18,4,100]	29.6	0.088	3.50	brush
[18,8,100]	77.6	0.065	0.60	comb
[18,16,100]	136	0.058	0.30	comb
[23,8,100]	69.3	0.077	0.82	comb
[41,8,100]	59.3	0.085	2.41	comb
PBA Brush PSAs				
[11,1,50]	42.6	0.212	0.90	brush
[11,1,100]	21.1	0.157	2.30	brush
[11,1,200]	8.47	0.098	15.2	brush
[11,2,200]	18.1	0.072	2.90	brush
[11,3,200]	31.6	0.067	1.40	brush
[11,10,200]	58.8	0.025	0.25	comb
[11,2,100]	37.6	0.132	0.85	brush
[23,2,100]	20.3	0.136	1.50	brush
[41,2,100]	6.69	0.142	3.21	brush

1) Structural code of brush elastomers defined by the DP of side chains (n_{sc}), backbone spacers between side chains (n_g), and the backbone between crosslinks (n_x). 2) Apparent Young's modulus (Eq. S2). 3) Strain stiffening parameter determined from fitting Eq. S1. 4) Rouse time determined from uniaxial tensile tests at various strain rates within $\dot{\varepsilon} = 10^{-4} - 10^{1}$ s^{-1} (Figures S25-29). 5) Bottlebrush (brush) and comb regimes identified depending on side chain length and grafting density[1].

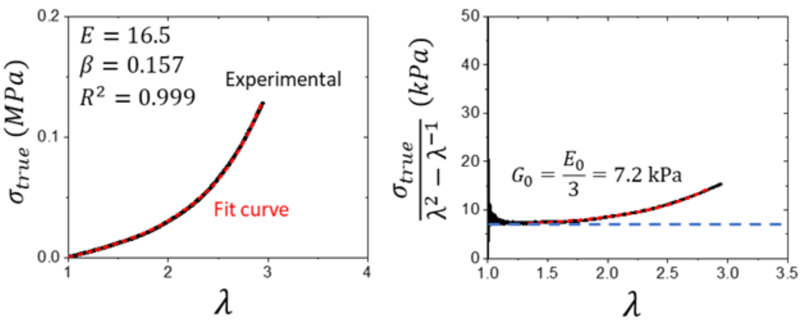

Method	$E_0(kPa)$
Fitting (current work)	21.1
Mooney-Rivlin	21.6

Figure S15. Comparing fitting vs. Mooney-Rivlin method for determination of E_0. The fitting method shows good agreement with the Mooney-Rivlin method within 3 percent variation. [(21.6kPa – 21.1kPa)/(21.6kPa)] * 100 = 2.3%. $\dot{\varepsilon}$ =0.005, T=20°C.

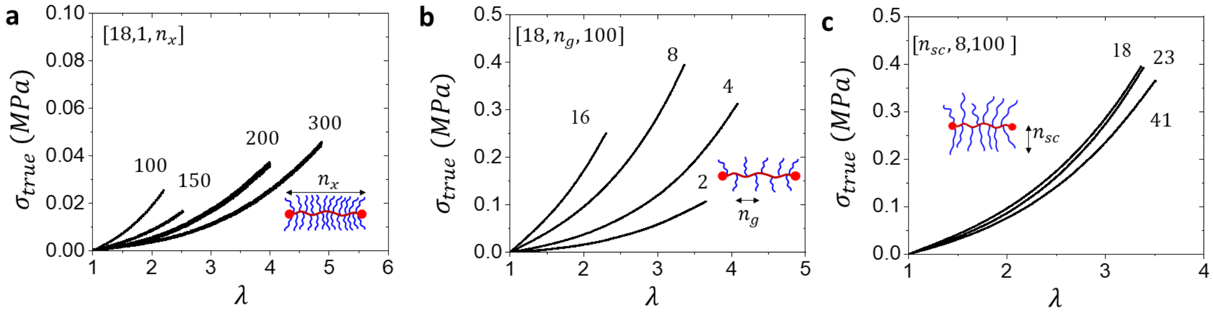

Figure S16. a) Stress-elongation curves at various n_x. As n_x increases, E_0 and β decrease. (n_g = 1, n_{sc} = 18, $\dot{\varepsilon}$ = 0.0001 s^{-1}). b) Stress-elongation curves at various n_g. The E_0 increases with n_g while β decreases. (n_x = 100, n_{sc} = 18). c) Stress-elongation curves at various n_{sc}. The E_0 decreases while n_{sc} and β increase. (n_g = 8, n_x =100). T=20°C. See E_0 and β values for each sample in Table S1.

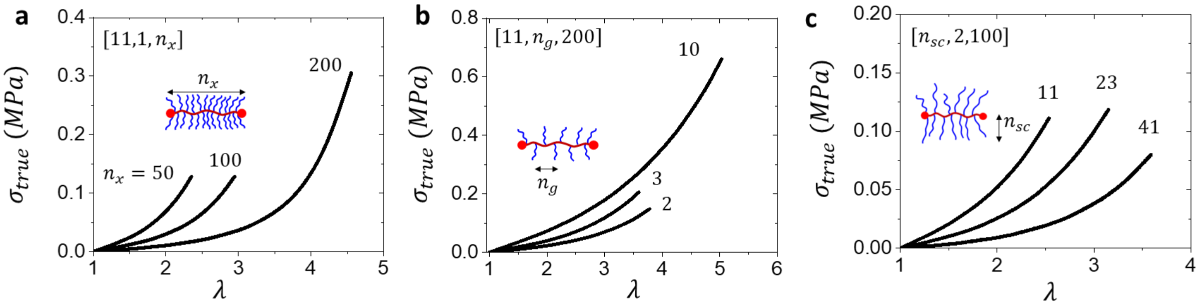

Figure S17. PBA brush elastomer mechanical analysis. a) Stress-elongation curves at various n_x. As n_x increases, E_0 and β decrease. (n_g = 1, n_{sc} = 11). b) Stress-strain curves at various n_g. The E_0 increases with n_g while β decreases. (n_x = 200, n_{sc} = 11). c) Stress-strain curves at various n_{sc}. The E_0 decreases while n_{sc} and β increase. (n_g = 2, n_x =100). T=20°C. See E_0 and β values for each sample in Table S1.

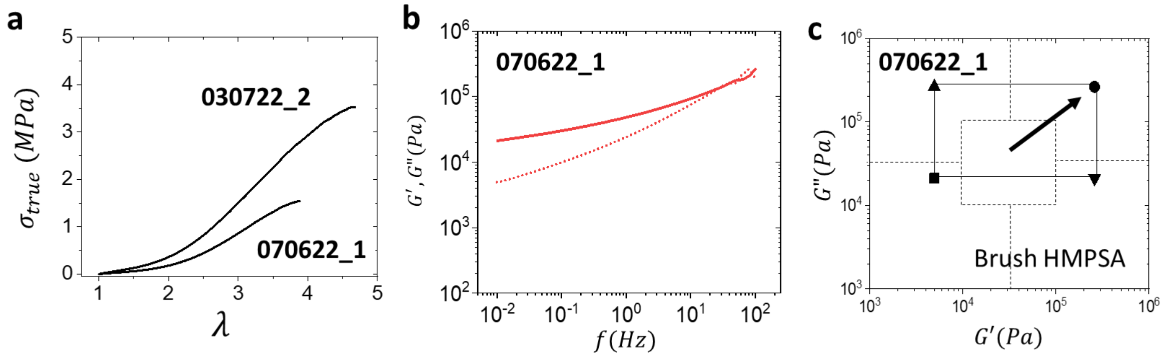

Figure S18. a) Stress-elongation curves at various A-g-B brush graft copolymer PSAs (Table S3). $\dot{\varepsilon}$=0.001s^{-1}, T=20°C. A-g-B brush copolymer are much stronger than the UV-cured elastomers. b) Frequency sweep of a sample A-g-B brush copolymer. c) Chang window for a sample A-g-B brush copolymer. The window shifts to the upper right indicating potential use as a high shear PSA.

Table S3. Mechanical properties of A-g-B brush copolymer PSAs.

Sample	n_g 1)	n_{sc}	n_x 2)	n_A 3)	ϕ_A 4)	n_{bb} 5)	E 6) (kPa)	β 7)	E_0 8) (kPa)	λ_{fit} 9)	λ_{max} 10)	σ_{max} 11) (kPa)
030722_2	8	18	216	60	0.108	1319	170	0.197	232	2.32	4.67	3520
070622_1	8	18	332	60	0.070	1237	97.2	0.174	127	2.80	3.88	1540

(1) Grafting density of side chains on the backbone with BA spacer. (2) Number average degree polymerization of brush backbone between glassy block side chains that physical crosslink. (3) Number average degree polymerization of each glassy block side chain as determined by ¹H-NMR. (4) Volume fraction glassy block, $\rho_{PIB} = 0.92\, g/mL$, $\rho_{PS} = 1.02\, g/mL$, $\rho_{PBA} = 1.08\, g/mL$. (5) Number average degree polymerization of the total brush strand. (6) Structural modulus $E \sim 1/\left(n_{bb}(n_{sc}+1)\right)$ and (7) strain-stiffening parameter $\beta = \langle R_{in}^2 \rangle / R_{max}^2$ are fitting parameters in equation S1. (8) Apparent Young's modulus determined either as tangent of a stress-strain curve at $\lambda \rightarrow 1$ or from the fitting equation S2. (9) Elongation range used for fitting equation S1 before deviation from the theory. (10) Maximum true stress and elongation at sample rupture. (11) Maximum stress-at-break (strength) of A-g-B brush copolymer samples.

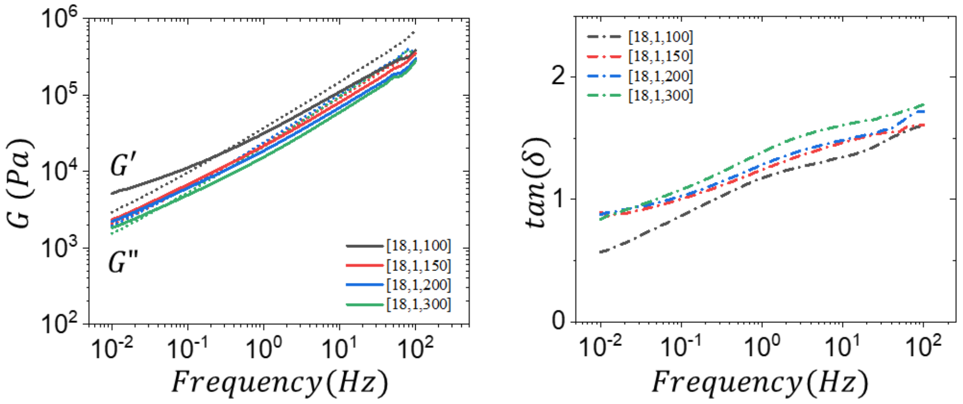

Figure S19. PIB viscoelastic control with n_x. As n_x increases, energy dissipation increases disproportionately resulting in greater tanδ. All bottlebrush PIB samples witness the proper balance between energy storage and dissipation with tanδ~1 within the frequency of the Chang window. T=20℃.

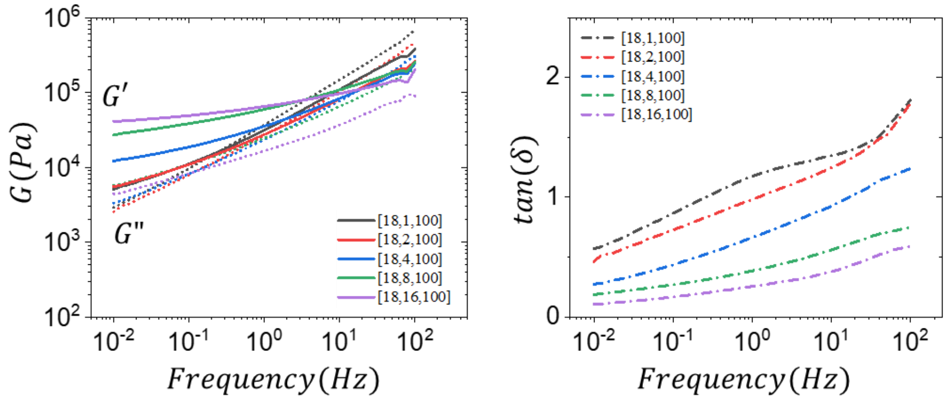

Figure S20. PIB viscoelastic control with n_g. As n_g increases, energy dissipation decreases disproportionately resulting in lower tanδ. Samples with greater grafting density of PIB macromonomer (i.e. $n_g = 1{,}2$) witness the proper balance between energy storage and dissipation with tanδ~1 within the frequency of the Chang window. T=20℃.

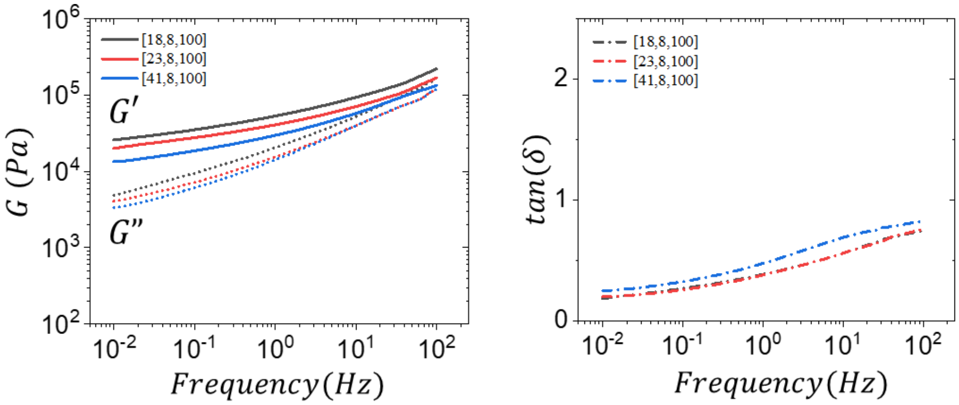

Figure S21. PIB viscoelastic control with n_{sc}. As n_{sc} increases, energy dissipation increases disproportionately resulting in greater tan δ. T=20℃.

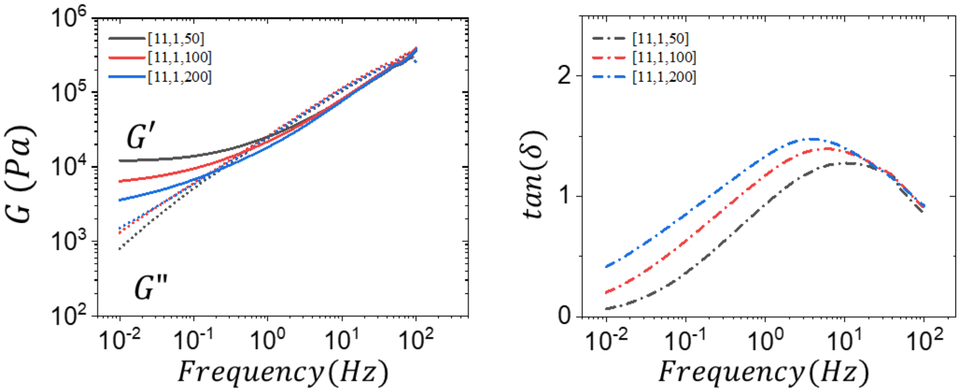

Figure S22. PBA viscoelastic control with n_x. As n_x increases, energy dissipation increases disproportionately resulting in greater tanδ. All bottlebrush PBA samples witness the proper balance between energy storage and dissipation with tanδ~1 within the frequency of the Chang window. T=20℃.

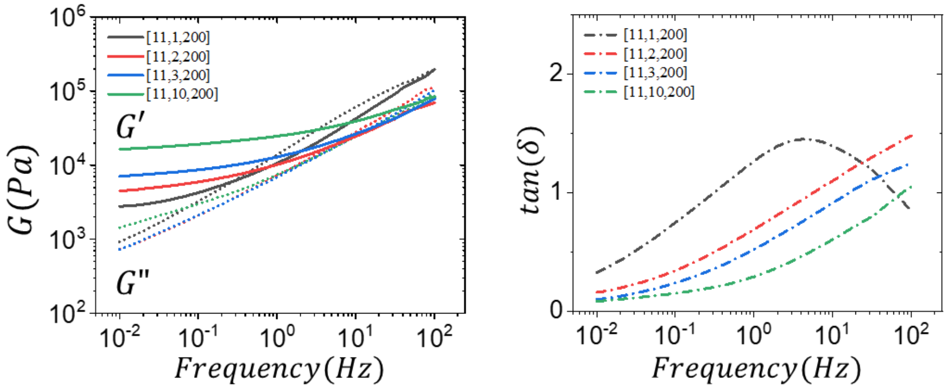

Figure S23. PBA viscoelastic control with n_g. As n_g increases, energy dissipation decreases disproportionately resulting in lower tanδ. Samples with greater grafting density of PBA macromonomer (i.e. $n_g = 1,2,3$) witness the proper balance between energy storage and dissipation with tanδ~1 within the frequency of the Chang window. T=20℃.

Figure S24. PBA viscoelastic control with n_{sc}. As n_{sc} increases, energy dissipation increases disproportionately resulting in greater tanδ. Brush PBA elastomer PSA samples witness the proper balance between energy storage and dissipation with tanδ~1 within the frequency of the Chang window. T=20℃.

5. Rouse time measurements

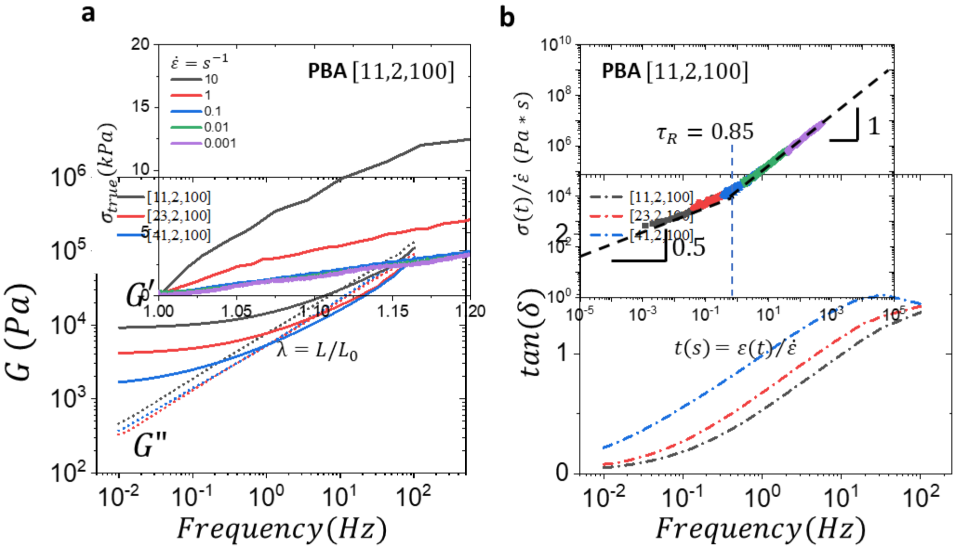

Figure S25. Example of Rouse time derivations from uniaxial tensile testing at various strain rates. a) Stress-elongation curves at various strain rates for a sample PBA brush PSA. b) Determining Rouse time from the slope-transition of rate normalized stress defining deformation in the Rouse regime (slope=0.5) to deformation on the elastic plateau (slope=1). T=20℃.

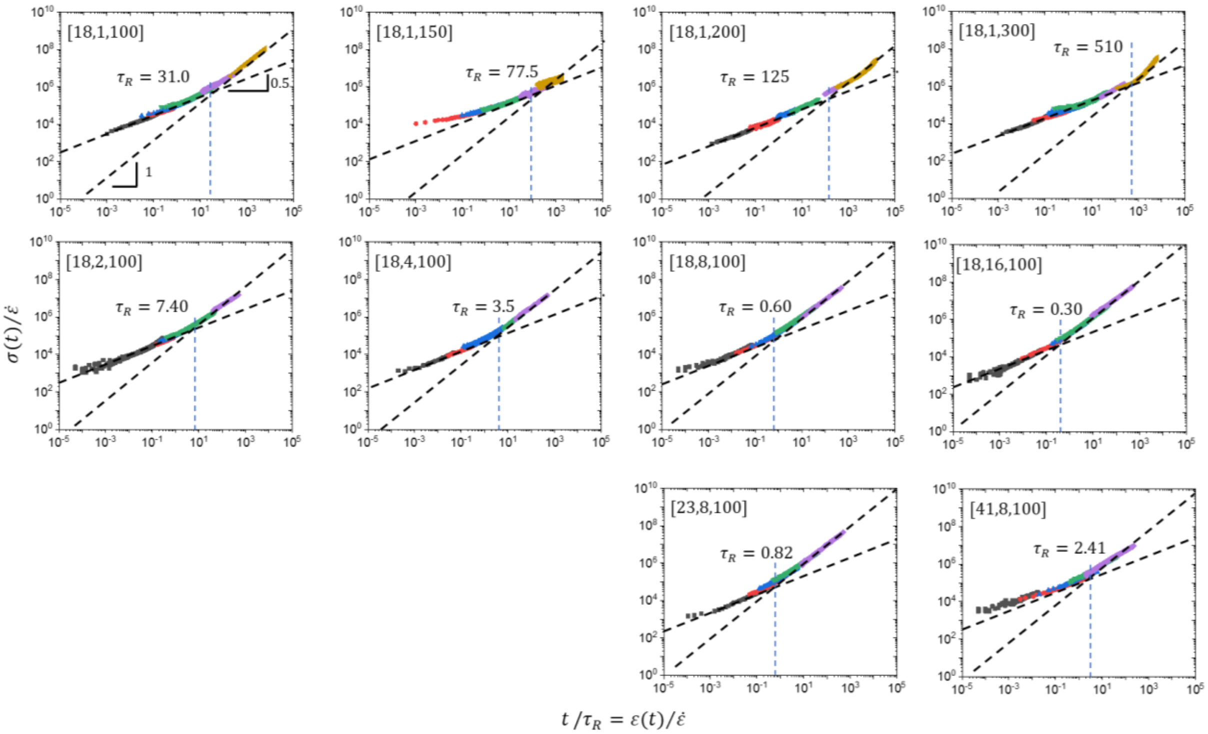

Figure S26. Stain rate normalized stress dependence on time for PIB brush PSA samples. The plots show a slope transition from 0.5 to 1 as the deformation of the sample transitions from the Rouse regime where the network can be approximated as a melt of polymer strands, to the elastic regime where contributions of crosslinks and entanglements must be considered. The τ_R of PIB brush elastomer PSAs was verified by differentiation and creation of a master curve in which all lines converge (Figures S28,29). T=20°C. 10mm/s (black), 1 mm/s (red), 0.1 mm/s (blue), 0.01 mm/s (green), 0.001 mm/s (purple), 0.0001 mm/s (yellow). Linear rates were translated to strain rates based on the initial length of the sample in the clamps.

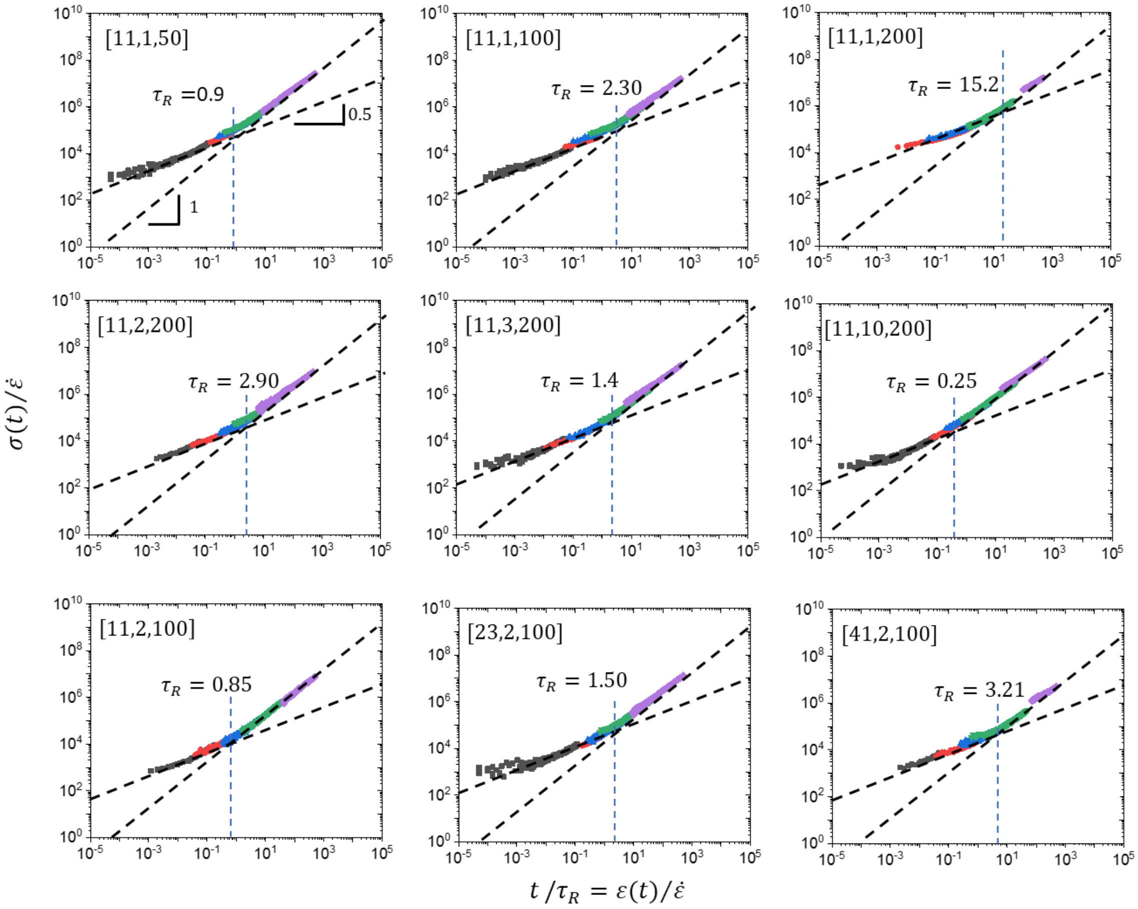

Figure S27. Stain rate normalized stress dependence on time for PBA brush PSA samples. The plots show a slope transition from 0.5 to 1 as the deformation of the sample transitions from the Rouse regime where the network can be approximated as a melt of polymer strands, to the elastic regime where contributions of crosslinks and entanglements must be considered. The τ_R of PBA brush elastomer PSAs was verified by differentiation and creation of a master curve in which all lines converge (Figures S28,29). T=20℃. 10mm/s (black), 1 mm/s (red), 0.1 mm/s (blue), 0.01 mm/s (green), 0.001 mm/s (purple), 0.0001 mm/s (yellow). Linear rates were translated to strain rates based on the initial length of the sample in the clamps.

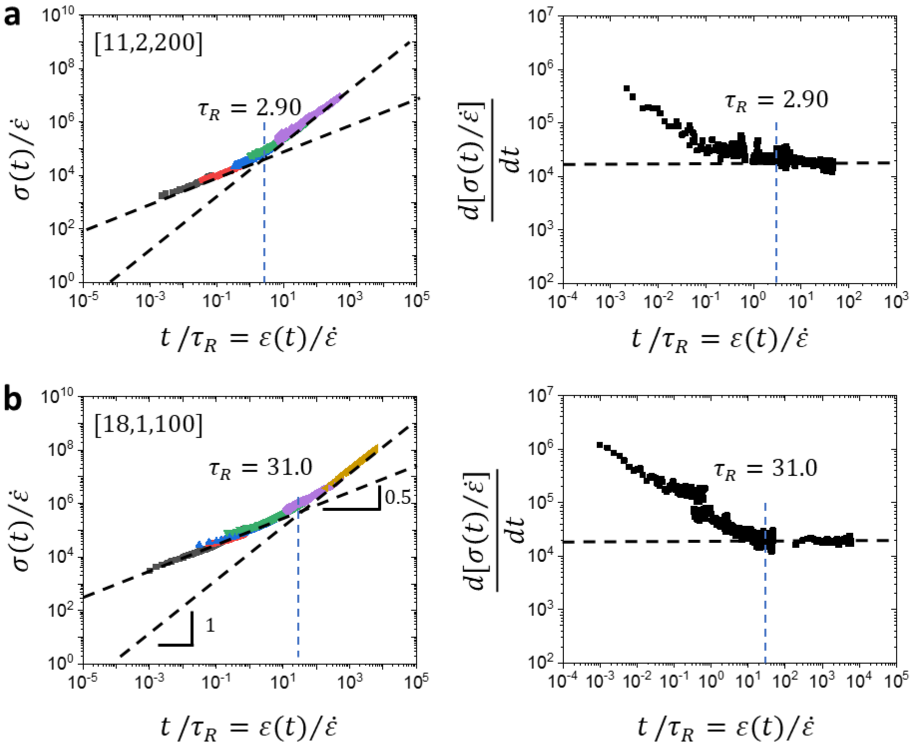

Figure S28. Verifying Rouse time estimation by differentiation. a) PBA brush elastomer [11,2,200]. b) PIB brush elastomer [18,1,100]. The derivatives level off at the estimated Rouse time. T=20℃.

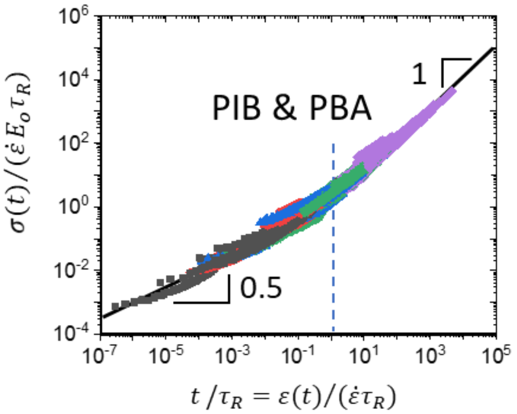

Figure S29. Universal curve to verify Rouse time estimation. Each sample collapses onto a single curve suggesting Rouse time estimates are precise compared to one another. T=20℃.

6. Work of adhesion measurements

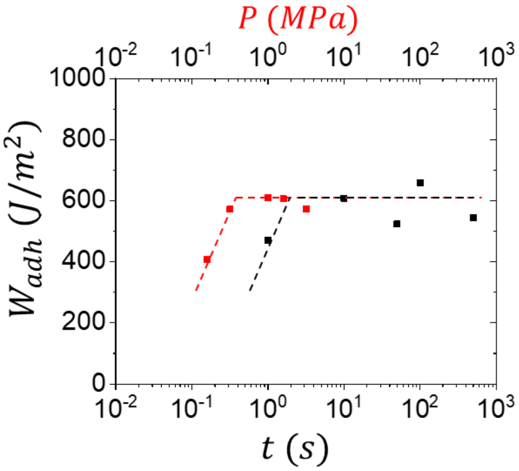

Figure S30. Optimization of contact pressure and dwelling time. The modified probe tack test was performed independently changing the contact pressure and contact time in effort to maximize the contact area of the PSA bond. The PIB brush elastomer PSA with architecture [18,1,300] was used because it had the greatest τ_R and would achieve the largest contact area at the interface. The contact area, represented through the W_{adh}, plateaued ~ 600 J/m^2. Parameters of 1 MPa contact pressure and 100s contact time were used for further experimentation. T=20°C.

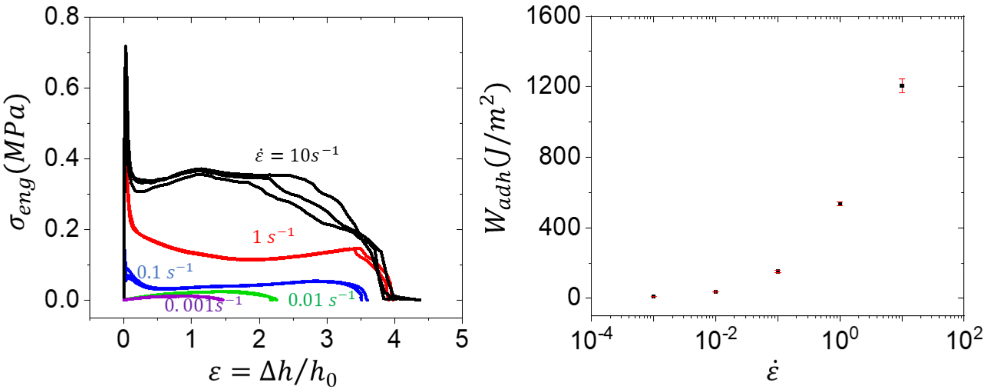

Figure S31. Repeatability of modified probe tack test experiments. Independent measurements were shown to be within 4% error (Table S4), where error bars are displayed in red on the plot to the right. PBA [11,1,200]. T=20°C.

Table S4. Precision of modified probe tack test with strain rate. Error was determined by standard deviation of triplicate measurements from Figure S31.

Sample	$\dot{\varepsilon}$ [(1)] (s^{-1})	W_{adh} [(2)] (J/m^2)	$\%Error$ [(3)]
PBA [11,1,200]	10	1165 ± 38.9	3.3
	1.0	537 ± 10.3	2.9
	0.1	151 ± 5.77	3.8
	0.01	37.0 ± 0.38	1.0
	0.001	11.3 ± 0.26	3.5

[(1)] Strain rate of debonding during the probe tack test. [(2)] Overall average work of adhesion for a desired debonding rate with relative uncertainty of a triplet of trials determined by standard deviation. [(3)] Representative present error for a given strain rate in determining W_{adh}. The percent error within the debonding strain rate range of 0.001-10 s^{-1} remains < 4% for the modified probe tack test used for all samples.

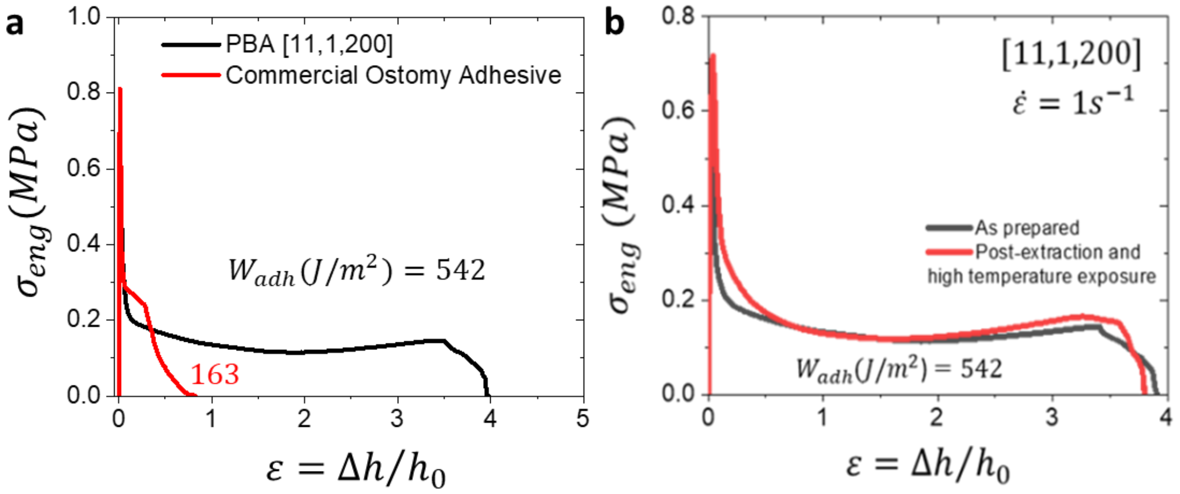

Figure S32. a) Brush PSAs display greater work of adhesion than a commercial ostomy bag and b) adhesion does not change after extraction in toluene and after exposure to high temperatures for a sample PBA brush PSA. T=20°C.

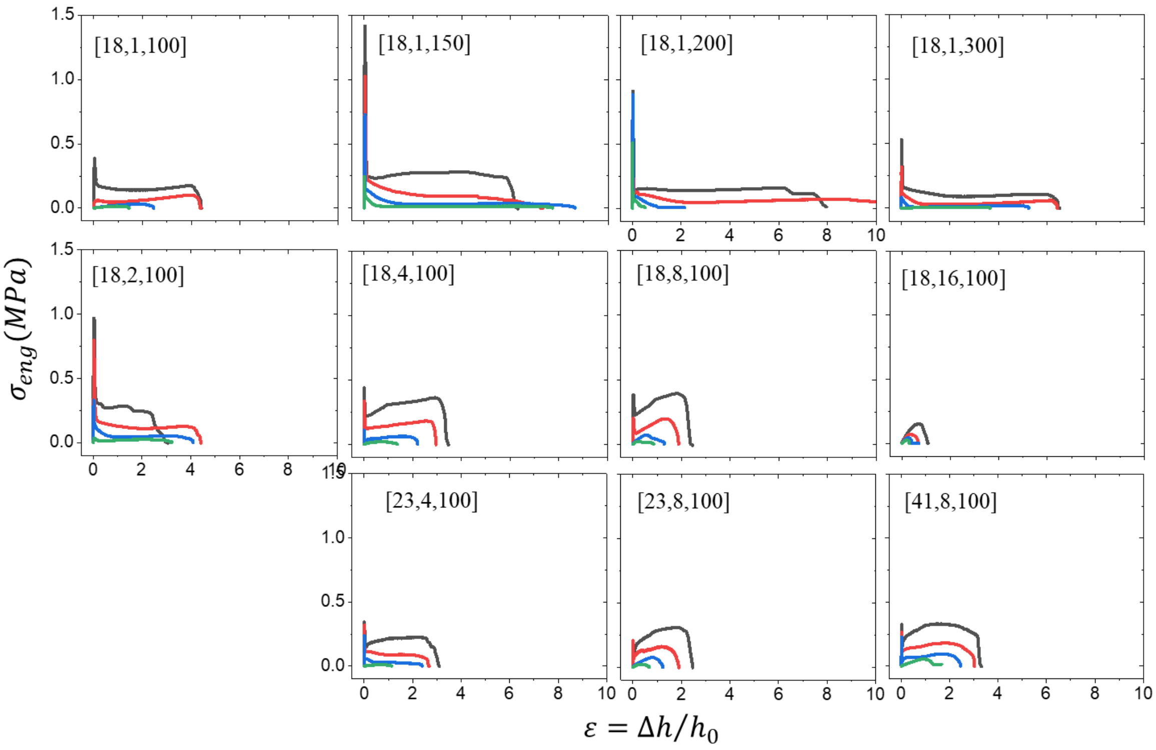

Figure S33. Raw modified probe tack test spectra for PIB brush PSAs. All PIB brush elastomer PSAs were subjected to modified probe tack testing at variable strain rates. As you increase n_x, fibril elongation before catastrophic failure of the adhesive bond increases though the stress from the fibrils decreased. Increasing the grafting density of the PIB side chains also increased the tack and fibril elongation. With increased n_{sc}, fibrillar stress increased and the fibril elongation increases minimally. T=20℃. Note: some samples were measured from $v = 10 - 0.01\ mm/s$ rather than $v = 1 - 0.001\ mm/s$, please see Table S5 to verify.

Table S5: Work of adhesion and strain rate dependence of PIB brush PSAs.

**Sample**	v (mm/s) (1)	$\dot{\varepsilon}$ (s^{-1}) (2)	W_{adh} (J/m^2) (3)
[18,1,100]	10^{0}	1.053	638.0
	10^{-1}	0.105	282.3
	10^{-2}	0.011	49.8
	10^{-3}	0.001	14.5
[18,1,150]	10^{1}	15.873	1035.1
	10^{0}	1.587	450.5
	10^{-1}	0.200	162.9
	10^{-2}	0.020	51.7
[18,1,200]	10^{1}	16.7	857
	10^{0}	1.67	432
	10^{-1}	0.167	43.2
	10^{-2}	0.017	13.2
[18,1,300]	10^{0}	1.124	609.5
	10^{-1}	0.112	242.3
	10^{-2}	0.011	72.9
	10^{-3}	0.001	18.6
[18,2,100]	10^{1}	10.417	706.3
	10^{0}	1.250	452.9
	10^{-1}	0.125	175.8
	10^{-2}	0.013	53.0
[18,4,100]	10^{1}	8.333	1250.4
	10^{0}	0.833	545.0
	10^{-1}	0.083	135.0
	10^{-2}	0.008	23.7
[18,8,100]	10^{1}	8.333	914.2

	10^{0}	0.833	330.2
	10^{-1}	0.083	66.1
	10^{-2}	0.008	14.6
[18,16,100]	10^{0}	0.870	242
	10^{-1}	0.087	50.7
	10^{-2}	0.009	5.6
	10^{-3}	0.001	0.7
[23,8,100]	10^{1}	11.111	605.0
	10^{0}	1.111	218.3
	10^{-1}	0.111	54.9
	10^{-2}	0.011	11.0
[41,8,100]	10^{0}	0.769	1075.1
	10^{-1}	0.077	527.8
	10^{-2}	0.008	210.1
	10^{-3}	0.001	58.0

(1) Linear velocity of debonding during the probe tack test of PIB brush elastomer PSAs. (2) Thickness normalized strain rate of debonding. (3) The overall work of adhesion determined from Eq. 2.

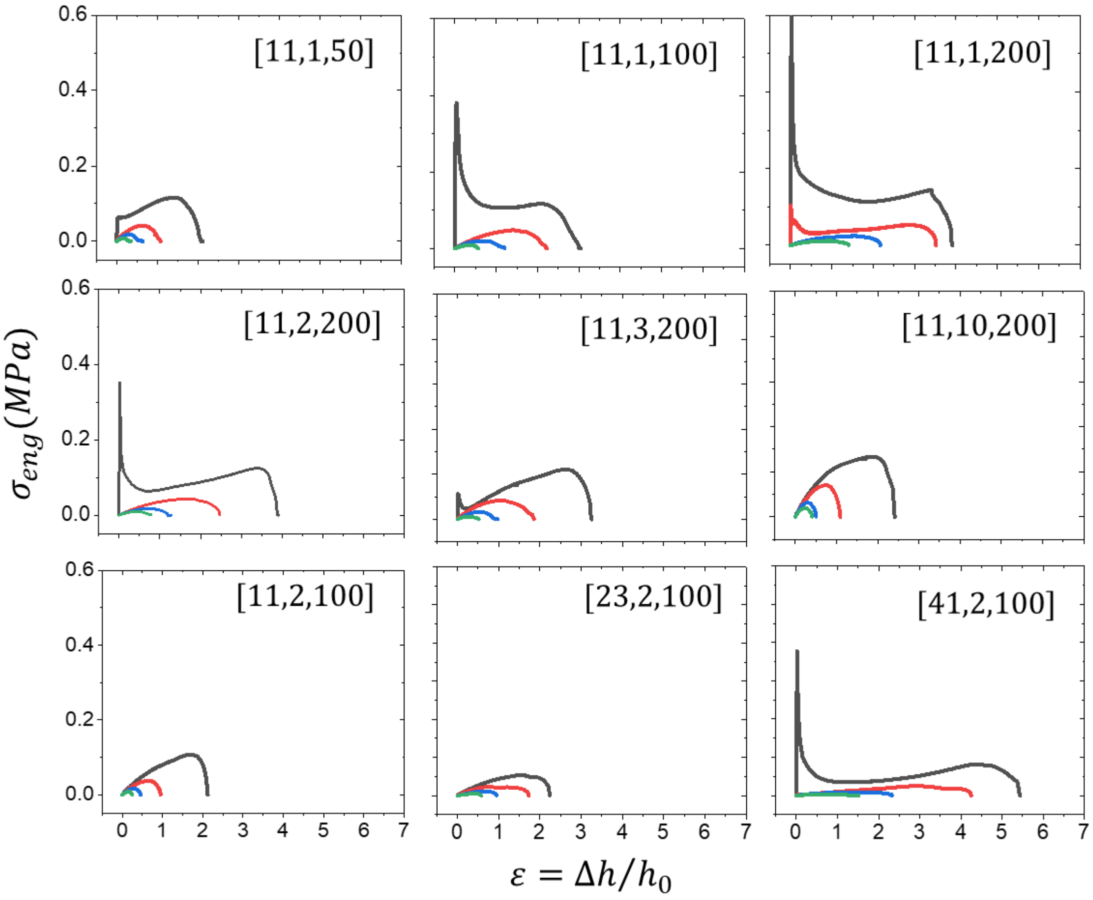

Figure S34. Raw modified probe tack test spectra for PBA brush PSAs. All PBA brush elastomer PSAs were subjected to modified probe tack testing at variable strain rates. As you increase n_x, fibril elongation before catastrophic failure of the adhesive bond and tack increase. Increasing the grafting density of the PBA side chains also increased the tack and fibril elongation. With increased n_{sc}, fibril elongation increases and tack peaks manifest. Note: all samples were measured from $v = 1 - 0.001\ mm/s$, 1mm/s (black), 0.1 mm/s (red), 0.01 mm/s (blue), 0.001 mm/s (green).

Table S6: Work of adhesion and strain rate dependence of PBA brush PSAs.

**Sample**	v $(mm/s)^{(1)}$	$\dot{\varepsilon}$ $(s^{-1})^{(2)}$	W_{adh} $(J/m^2)^{(3)}$
[11,1,50]	10^{0}	0.867	207.7
	10^{-1}	0.087	34.6
	10^{-2}	0.009	7.5
	10^{-3}	0.001	1.9
[11,1,100]	10^{0}	0.909	377.5
	10^{-1}	0.091	76.5
	10^{-2}	0.009	16.9
	10^{-3}	0.001	3.6
[11,1,200]	10^{0}	1.000	527.3
	10^{-1}	0.100	148.4
	10^{-2}	0.010	37.4
	10^{-3}	0.001	11.3
[11,2,200]	10^{0}	0.862	416.1
	10^{-1}	0.086	86.0
	10^{-2}	0.009	16.4
	10^{-3}	0.001	5.7
[11,3,200]	10^{0}	0.833	286.4
	10^{-1}	0.083	63.5
	10^{-2}	0.008	11.9
	10^{-3}	0.001	2.4
[11,10,200]	10^{0}	0.833	160
	10^{-1}	0.083	36.5
	10^{-2}	0.008	14.6
	10^{-3}	0.0001	2.3

[11,2,100]	10^{0}	0.800	186.7
	10^{-1}	0.080	30.4
	10^{-2}	0.008	6.2
	10^{-3}	0.001	1.7
[23,2,100]	10^{0}	0.833	1208
	10^{-1}	0.083	34.5
	10^{-2}	0.008	8.5
	10^{-3}	0.001	1.7
[41,2,100]	10^{0}	1.052	296.3
	10^{-1}	0.105	61.3
	10^{-2}	0.011	13.2
	10^{-3}	0.001	2.9

(1) Linear velocity of debonding during the probe tack test of PBA brush elastomer PSAs. (2) Thickness normalized strain rate of debonding. (3) The overall work of adhesion determined from Eq. 2.

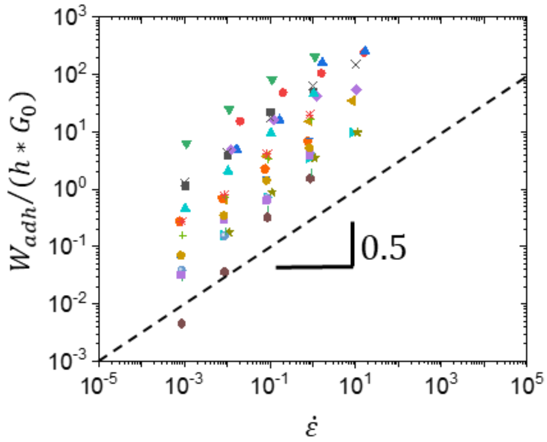

Figure S35. Normalized work of adhesion before experimental Rouse time shift with architecture compared to Figure 3d. Changing architecture alone yields ~4-decade shift in the normalized work of adhesion for a given strain rate. All samples show a concurrent increase in normalized work of adhesion with strain rate. Scaling for each sample depends on relaxation dynamics elicited in Figure 3d,e.

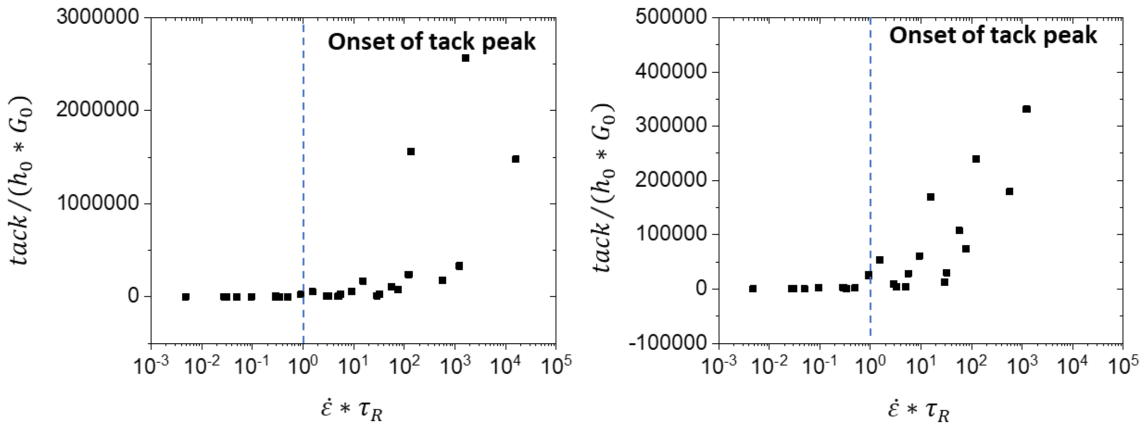

Figure S36. Onset of viscoelastic debonding mechanism defined by manifestation of the tack peak. This is of a subset of PIB brush PSAs where tack = σ_{tack}.

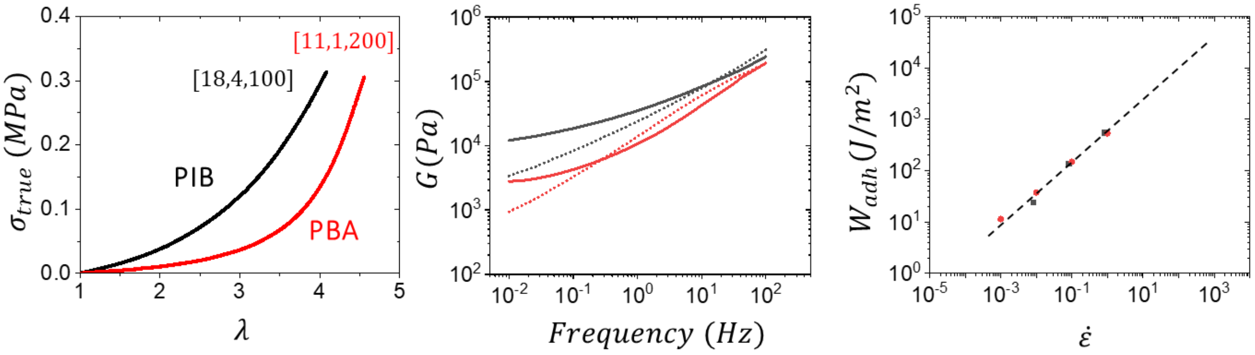

Figure S37. Different and chemistry, same work of adhesion. From left to right: uniaxial tensile tests display different equilibrium mechanical properties of softness and firmness (Table S1). Their rheological curves also vary greatly within the Chang window. However, the work of adhesion is nearly identical through four decades of strain rate. This can be attributed to different modes of debonding and resistance to deformation as well as the difference in thermodynamic work of adhesion between the adhesive and probe. T=20°C.

7. Theoretical Analysis

Rouse time of brush elastomer PSA's.

To elucidate the dependence of the Rouse time of bottlebrush polymers on grafting density and degree of polymerization of the side chains, we assume that two bottlebrushes contact each other at the surface of their perspective side chain blobs due to expulsion of the side chains and backbones. This results in the macromolecular friction coefficient being proportional to the number of monomers on the outer surface of bottlebrushes. The surface area of the bottlebrush (Figure S38) with backbone degree of polymerization n_x is estimated as

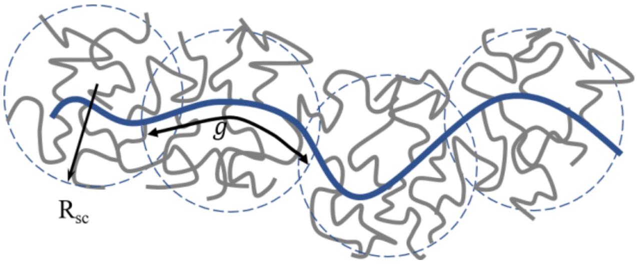

Figure S38. A brush macromolecule as a chain of blobs with size R_{sc} each containing g backbone monomers.

$$A \approx n_x R_{sc}^2/g \tag{S7}$$

where R_{sc} is the side chain size and g is the number of backbone monomers per side chain thickness. Taking into account the packing condition of monomers having excluded volume v

$$g\left(1 + n_{sc}/n_g\right)v/R_{sc}^3 \approx 1 \rightarrow R_{sc}^2/g \approx v\varphi^{-1}/R_{sc} \tag{S8}$$

the total number of monomers on the surface of the brush is

$$n_{surf} \approx A/v^{2/3} \approx n_x\, v^{1/3}\varphi^{-1}/R_{sc}. \tag{S9}$$

The corresponding friction coefficient of the bottlebrush with monomer friction coefficient ζ_0 is equal to

$$\zeta_n = \zeta_0 n_{surf} \approx \zeta_0\, n_x\, v^{1/3}\varphi^{-1}/R_{sc} \tag{S10}$$

The Rouse time of the brush macromolecule with size

$$R^2 \approx n_x R_{sc}^2/g \approx n_x v\varphi^{-1}/R_{sc} \tag{S11}$$

is estimated as

$$\tau_R \approx \zeta_n R^2/k_BT \approx \zeta_0 v^{4/3}(n_x\varphi^{-1})^2/k_BTR_{sc}^2 \tag{S12}$$

For brushes with stretched backbones and ideal side chains ($R_{sc}^2 \approx lbn_{sc}$) corresponding to the SBB regime, eq S12 is rewritten as follows

$$\tau_R \approx \tau_0(n_x\varphi^{-1})^2/n_{sc} \tag{S13}$$

where $\tau_0 \approx \zeta_0 v^{4/3}/lbk_BT$ is a characteristic relaxation time of brushes with monomer projection length l and Kuhn length b. Note that a peculiar dependence of τ_0 on polymer chemistry specific parameters v, l and b is due to brush friction mechanisms and side chain packing constraints. It is different from that of linear chains in a melt for which a characteristic relaxation time $\tau_{0,lin} \approx \zeta_0 lb/k_BT$.

For brush strands with densely grafted side chains $\varphi^{-1} \approx n_{sc}/n_g$, we obtain the following dependence of the Rouse time on brush architecture (main text, Eq. 1),

$$\tau_R \approx \tau_0 n_{sc}\left(\frac{n_x}{n_g}\right)^2 \propto n_{sc}\left(\frac{n_x}{n_g}\right)^2. \tag{S14}$$

It is important to point out that we will have a different dependence of the Rouse time on the molecular architecture if brush friction is determined by all monomers. In this case, the net friction coefficient is equal to

$$\zeta_n = \zeta_0 n_x\varphi^{-1} \tag{S15}$$

and the corresponding Rouse time is

$$\tau_R \approx \zeta_n R^2/k_BT \approx \zeta_0(n_x\varphi^{-1})^2 v/k_BTR_{sc} \tag{S16}$$

resulting in a stronger dependence of the strand relaxation time on the side chain DP in the SBB regime

$$\tau_R \propto n_{sc}^{3/2} \left(\frac{n_x}{n_g}\right)^2. \tag{S17}$$

8. Videos

Movie S1. Probe tack tests of PBA brush PSAs with different side chain DP denoted by $[\boldsymbol{n_{sc}}, \boldsymbol{n_g}]$. Samples with increased side chain length resulted in greater work of adhesion and fibril elongation. This is a video representation in coordination with viscoelasticity shown in Figure 3a.

Movie S2. Hanging weight tests of PIB brush PSAs with different grafting density denoted by $[\boldsymbol{n_{sc}}, \boldsymbol{n_g}]$. Samples with increased grafting density resulted in being able to uphold larger loads. This is a video representation of Figure 3c where the hanging weight apparatus is 12 lb/in^2 itself.

Movie S3. PBA and PIB brush PSAs with the same modulus ($\boldsymbol{E_0}$~30 kPa) and strain-stiffening ($\boldsymbol{\beta \sim 0.08}$) during probe tack testing. This acts as a visual representation of the quantitative observations denoted in Figure 4a. The PIB brush PSA sample has greater work of adhesion and tack.

Movie S4. PIB and PBA brush PSA samples with different equilibrium mechanical properties but the same work of adhesion from strain rates 0.001 to 1s^{-1}. This video is a visual representation of data in Figure S36 at $\boldsymbol{\dot{\varepsilon} \sim 1 s^{-1}}$.

Movie S5. PBA brush PSAs with the same modulus ($\boldsymbol{E_0}$~20 kPa) but different strain stiffening ($\boldsymbol{\beta}$) debonding from a large probe. This acts as a visual representation of the quantitative observations denoted in Figure 4b. The larger $\boldsymbol{\beta}$ sample detaches before the sample with lower $\boldsymbol{\beta}$. Samples were deboned at $\boldsymbol{\dot{\varepsilon} \sim 1 s^{-1}}$.

9. References

(1) Maw, M.; Morgan, B. J.; Dashtimoghadam, E.; Tian, Y.; Bersenev, E. A.; Maryasevskaya, A. V.; Ivanov, D. A.; Matyjaszewski, K.; Dobrynin, A. V.; Sheiko, S. S. Brush architecture and network elasticity: path to the design of mechanically diverse elastomers. *Macromolecules* **2022**, *55* (7), 2940–2951. https://doi.org/10.1021/acs.macromol.2c00006.

(2) Dobrynin, A. V.; Carrillo, J.-M. Y. Universality in nonlinear elasticity of biological and polymeric networks and gels. *Macromolecules* **2011**, *44* (1), 140–146. https://doi.org/10.1021/ma102154u.

(3) Cao, Z.; Daniel, W. F. M.; Vatankhah-Varnosfaderani, M.; Sheiko, S. S.; Dobrynin, A. V. Dynamics of bottlebrush networks. *Macromolecules* **2016**, *49* (20), 8009–8017. https://doi.org/10.1021/acs.macromol.6b01358.

(4) Ward, I. M.; Sweeney, J. *Mechanical Properties of Solid Polymers*; John Wiley & Sons, 2012.

(5) Larson, R. G. *Constitutive Equations for Polymer Melts and Solutions: Butterworths Series in Chemical Engineering*; Butterworth-Heinemann, 2013.

(6) Lakrout, H.; Sergot, P.; Creton, C. Direct observation of cavitation and fibrillation in a probe tack experiment on model acrylic pressure-sensitive-adhesives. *J. Adhes.* **1999**, *69* (3–4), 307–359. https://doi.org/10.1080/00218469908017233.

(7) Sztucki, M.; Narayanan, T. Development of an ultra-small-angle X-ray scattering instrument for probing the microstructure and the dynamics of soft matter. *J. Appl. Crystallogr.* **2007**, *40* (s1), s459–s462. https://doi.org/10.1107/S0021889806045833.
(8) Kline, S. R. Reduction and analysis of SANS and USANS data using IGOR Pro. *J. Appl. Crystallogr.* **2006**, *39* (6), 895–900. https://doi.org/10.1107/S0021889806035059.
(9) Dashtimoghadam, E.; Maw, M.; Keith, A. N.; Vashahi, F.; Kempkes, V.; Gordievskaya, Y. D.; Kramarenko, E. Y.; Bersenev, E. A.; Nikitina, E. A.; Ivanov, D. A.; Tian, Y.; Dobrynin, A. V.; Vatankhah-Varnosfaderani, M.; Sheiko, S. S. Super-soft, firm, and strong elastomers toward replication of tissue viscoelastic response. *Mater. Horiz.* **2022**. https://doi.org/10.1039/D2MH00844K.
